# Sex‐based immune microenvironmental feature heterogeneity in response to PD‐1 blockade in combination with chemotherapy for patients with untreated advanced non‐small‐cell lung cancer

**DOI:** 10.1002/cam4.7423

**Published:** 2024-06-20

**Authors:** Xiaofeng Yu, Zhaolei You, Ying Liu, Jian Fang, Qi Zhao, Zhihong Sun, Yingjian Song, Jie Liu, Chengming Sun

**Affiliations:** ^1^ Department of Thoracic Surgery Yantai Yuhuangding Hospital Yantai China; ^2^ Department of Medical Oncology Yantai Yuhuangding Hospital Yantai China; ^3^ Department of Clinical Laboratory Yantai Yuhuangding Hospital Yantai China

**Keywords:** efficacy, NSCLC, PD‐1, sex, tumor immune microenvironment

## Abstract

**Background:**

To investigate the sex‐based heterogeneity of immune microenvironmental feature and its impact on the response to first‐line PD‐1 blockade plus chemotherapy in patients with driver‐negative advanced or metastatic non‐small‐cell lung cancer (NSCLC).

**Patients and Methods:**

A total of 439 patients with advanced NSCLC treated with first‐line PD‐1 blockade plus chemotherapy or chemotherapy were identified. Differences in clinical outcomes between female and male patients were determined using Kaplan–Meier curves. Neoantigen burden and five immune microenvironmental markers expression including PD‐L1, CD4, CD8, FOXP3, and CD68 were compared between two groups.

**Results:**

Of 175 eligible patients, 89 received PD‐1 blockade plus chemotherapy and 86 received first‐line chemotherapy. Forty five were women (25.7%) and 130 were men (74.3%). Female patients received first‐line PD‐1 blockade in combination with chemotherapy had dramatically better ORR (85.2% vs. 53.2%; *p* = 0.009), PFS (23.7 vs. 7.3 months; *p* = 0.013), and OS (46.2 vs. 20.0 months; *p* = 0.004) than males. Treatment outcomes were similar between females and males in chemotherapy group. Multivariate analyses showed that sex was the independent prognostic factor for patients received PD‐1 blockade combined with chemotherapy. Although female patients had significantly lower tumor mutational and neoantigen burden than males, pretreatment tumor tissues of female patients had markedly higher CD4, CD4/FOXP3, and CD4/FOXP3/PD‐L1 expression level than male patients.

**Conclusions:**

Female patients with untreated advanced or metastatic NSCLC would derive a larger benefit from PD‐1 blockade in combination with chemotherapy than males. The biological significances of heterogeneity of tumor immune microenvironmental features between them need further investigation.

## Graphical abstract

Although sex could affect the adaptive antitumor immunity, whether it had impact on the efficacy of first‐line PD‐1 blockade plus chemotherapy in advanced NSCLC remained undetermined. This study reported that female patients with advanced NSCLC would derive a larger benefit from first‐line PD‐1 blockade plus chemotherapy than male patients. Pretreatment tumor tissues from female patients had markedly lower tumor mutation and neoantigen burden, but significantly higher CD4, CD4/FOXP3, and CD4/FOXP3/PD‐L1 expression level than male patients, suggesting sex‐based neoantigen burden and immune microenvironmental feature heterogeneity would orchestrate the response to first‐line PD‐1 blockade plus chemotherapy in advanced NSCLC.

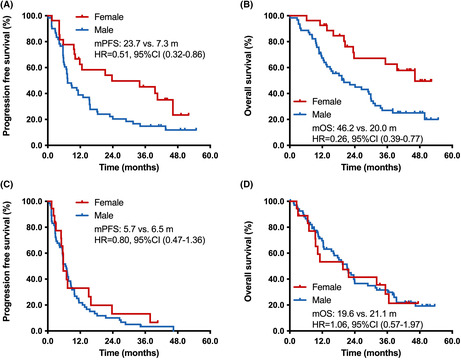

## 1 INTRODUCTION

Non‐small‐cell lung cancer (NSCLC) is the major histology of lung cancers and remains the major cause of cancer‐related death worldwide.^1^ Recently, antibodies targeting programmed cell death 1 (PD‐1) or its ligand (PD‐L1) have shifted the treatment paradigm of advanced NSCLC without driver gene alterations.^1^, ^2^, ^3^ Several elegant phase III randomized clinical trials have shown that first‐line PD‐1 blockade plus chemotherapy could dramatically improve the progression‐free survival (PFS) and/or overall survival (OS) in patients with advanced NSCLC.^4^, ^5^, ^6^, ^7^, ^8^, ^9^, ^10^, ^11^ However, not all of patients with advanced NSCLC can benefit from this combination regimen.^1^ Currently, only three officially approved biomarkers were used to guide the clinical application of PD‐1 antibody monotherapy, including PD‐L1 expression, microsatellite instability (MSI) status and tumor mutational burden (TMB),^12^ with moderate predictive performance. Nevertheless, they were failed to predict the response to PD‐1 blockade combined with chemotherapy. Under these circumstances, using clinicopathologic features to select the population who can benefit more from first‐line PD‐1 blockade plus chemotherapy is an economical and practical approach.

As one of the significant clinicopathologic parameters, sex plays an important role in the tumorigenesis and progression of nonreproductive organ cancers.^13^, ^14^ The complex interactions between sexual hormones, genes, diet, the environmental and tumor‐intrinsic factors could result in the sex‐related difference of cancer incidence and severity.^15^, ^16^ Recently, several studies reported that sex differences in tumor burden and treatment response mainly dependent on the adaptive immunity, especially CD8^+^ T cell‐mediate antitumor immunity.^17^, ^18^, ^19^ Several meta‐analyses of high‐quality clinical trials showed the magnitude of immune checkpoint inhibitors (ICIs) monotherapy benefit is sex‐dependent and women with advanced cancers seem to derive a larger benefit, but the results were inconsistent.^20^, ^21^, ^22^, ^23^, ^24^ To date, PD‐1 blockade plus chemotherapy has become the standard first‐line setting for many solid tumors including advanced NSCLC, esophageal, gastric, nasopharyngeal cancer, etc. Given the addition of chemotherapy would increase tumor cell death associated antigen release and elimination of some immunosuppressive cells, such as myeloid‐derived suppressor cell (MDSCs), regulatory T cells (Tregs) and so on,^25^, ^26^, ^27^ whether sex had impact on the efficacy of PD‐1 blockade plus chemotherapy in untreated advanced NSCLC remained undetermined.

Considering the significance of sex on antitumor immunity and unclear biomarkers for PD‐1 blockade in combination with chemotherapy, this study aimed to investigate the impact of sex on the efficacy of first‐line PD‐1 blockade in combination with chemotherapy or chemotherapy alone for patients with advanced or metastatic NSCLC. To unravel the potential biological explanations on the sex‐base heterogeneity in treatment response, we also surveyed the neoantigen burden and expression level of several tumor immune microenvironmental related markers tested by multiplex immunofluorescence (mIF) assays in pretreatment tumor tissue samples, including PD‐L1, CD8, CD4, FOXP3, CD68, and their biological combinations, between male and female patients.

## 2 MATERIALS AND METHODS

### 2.1 Patients' cohort

Patients pathologically diagnosed with advanced or metastatic NSCLC treated with first‐line PD‐1 blockade plus chemotherapy or chemotherapy alone were retrospectively identified in our center from February 1, 2016 to December 1, 2022. In brief, patients met the following criteria were eligible: aged 18–80 years old, Eastern Cooperative Oncology Group performance status (ECOG PS) of 0 or 1, pathologically confirmed stage IIIB–IV NSCLC without driver gene alterations (including *EGFR*, *ALK*, and *ROS1* alterations), first‐line PD‐1 blockade plus chemotherapy or chemotherapy alone, at least one measurable lesion per Response Evaluation Criteria in Solid Tumors version 1.1 (RECIST v1.1). For patients with non‐squamous NSCLC, first‐line chemotherapy regimen is carboplatin (area under curve [AUC], 5 mg/mL/min) plus pemetrexed (500 mg/m^2^) on Day 1 of each 3‐week treatment cycle for 4–6 cycles, followed by maintenance therapy with pemetrexed alone until disease progression, unacceptable toxicity, death, or physician decision. For patients with squamous NSCLC, first‐line chemotherapy regimen is carboplatin (AUC, 5 mg/mL/min) plus paclitaxel (175 mg/m^2^) on Day 1 of each 3‐week treatment cycle for 4–6 cycles. The PD‐1/PD‐L1 antibodies used in this study included pembrolizumab (200 mg), camrelizumab (200 mg), and tislelizumab (200 mg). These antibodies were given as maintenance therapy in combination with pemetrexed for patients with non‐squamous NSCLC or alone for patients with squamous NSCLC until disease progression, unacceptable toxicity, death, or physician decision. The maximum duration of PD‐1/PD‐L1 antibodies exposure was 2 years. The dose of all antitumor drugs was leveraged according to the recommended dose from drug instructions or predefined protocol of trials. The study protocol was conducted according to the local laws and regulations of China and Declaration of Helsinki, Guidelines for Good Clinical Practice. It was reviewed and approved by the ethics committee and institutional review board of our center.

### 2.2 Data collection

We collected these major clinicopathological characteristics including age, sex, smoking history, ECOG PS, histological types, number and sites of metastasis, PD‐L1 expression level, and therapeutic regimens. Age, smoking status, and ECOG PS were recorded at initial diagnosis. A person who had smoked <400 packs‐year during his/her lifetime was defined as never smoker. PD‐L1 expression level was tested by the DAKO 22C3 immunohistochemical staining assay according to the manufactory's instruction. Positive expression was defined according to the manufacturer's recommendations. Treatment response including complete response (CR), partial response (PR), stable disease (SD), and disease progression (PD) was evaluated using RECIST v1.1. PFS was calculated from the date of first‐line treatment initiation to the date of systemic progression or death or was censored at the date of the last tumor assessment (when performed). OS was defined as the interval from the date of the initial diagnosis to death from any reasons or was censored at the last follow‐up date. Data of eligible patients from electronic medical records were collected by using the same requirements for clinical data. Last follow‐up was April 1, 2023.

### 2.3 Tumor mutation and neoantigen burden calculation

Fresh or archival formalin fixed paraffin‐embedded (FFPE) tumor tissue samples at baseline were collected. Fresh biopsy tumor tissues were snap‐frozen in liquid nitrogen within 30 min. Ten milliliter peripheral blood samples were collected before any systemic antitumor treatment in ethylene diamine tetraacetic acid‐coated tubes (BD Biosciences, Franklin Lakes, USA). White blood cells were separated by centrifuging at 1800*g* for 10 min within 2 h of collection of blood samples. Genomic DNA from tumor tissues and white blood cells were extracted by using the DNeasy Blood and Tissue Kit (Qiagen, GmBH, Germany), QIAamp DNA FFPE Tissue Kit (Qiagen, GmBH, Germany), and Tguide S32 Magnetic Blood Genomic DNA Kit (TIANGEN, China) according to manufacturers' protocols. DNA concentration was assessed by Qubit dsDNA High Sensitivity Assay Kit (Thermo Fisher, USA) and the quality of DNA was measured by Agilent 2100 BioAnalyzer (Agilent, USA). Details of next‐generation sequencing and bioinformatic analyses were summarized in Supplemental Materials (Online methods).

### 2.4 Tumor immune microenvironmental features analysis

Pretreatment fresh or archival FFPE samples within 1 month before firs‐line setting were collected. PD‐L1, CD8, CD4, FOXP3, and CD68 were tested using the Opal 7‐Color fIHC Kit (Akoya Biosciences, Marlborough, USA) by manual mIF staining conducted in 4‐μm sequential histological tumor sections. Briefly, the prepared slides were deparaffinized, rehydrated, and subjected to epitope retrieval by boiling in Tris‐EDTA buffer at 97°C for 20 min. Endogenous peroxidases were then blocked by incubation in Antibody Diluent/Block for 10 min. In each round, only one antigen was tested including primary/secondary antibody incubation, TSA visualization, followed by labeling the next antibody after epitope retrieval and protein blocking. The antigens were successively tested in this order: FOXP3, CD8, PD‐L1, CD4, and CD68. Finally, 4′, 6‐Diamidino‐2‐Phenylindole were leveraged to stain the nuclei and mounted with anti‐quenching sealing tablets. The human tonsil tissues with and without primary antibodies were used as the positive and negative controls, respectively. Vectra multispectral microscope (Akoya Biosciences) were used to scan the slides. Two senior experienced pathologists independently tested the multispectral images by using the InForm 3.0 software for tissue segmentation (Akoya Biosciences). More details were summarized in the Supplemental Materials.

### 2.5 Statistical analyses

The categorical variables were analyzed by using chi‐squared or Fisher's exact test and the continuous variables were analyzed by using ANOVA and Tukey's multiple comparison tests. Mann–Whitney *U* tests or Kruskal–Wallis rank‐sum tests were used to analyze the continuous variables across multiple groups. Comparsions of each immune microenvironmental marker expression levels between female and male patients were conducted using nonparametric test. The corresponding 95% confidence intervals (CIs) of ORR and DCR were estimated using the stratified Cochran‐Mantel‐Haenszel method. The median survival time of PFS and OS were estimated using the Kaplan–Meier curves. Between‐group comparisons in PFS and OS were evaluated using a stratified log‐rank test. The Cox proportional hazards model and the hazard ratios (HRs) and corresponding 95% CIs were calculated and recorded for uni‐ and multivariate survival analyses. All statistical analyses were conducted using GraphPad PRISM 9.0 (GraphPad Software, San Diego, USA). *p* < 0.05 (two‐side) was statistically significant.

## 3 RESULTS

### 3.1 Patients' characteristics

**FIGURE 1:**
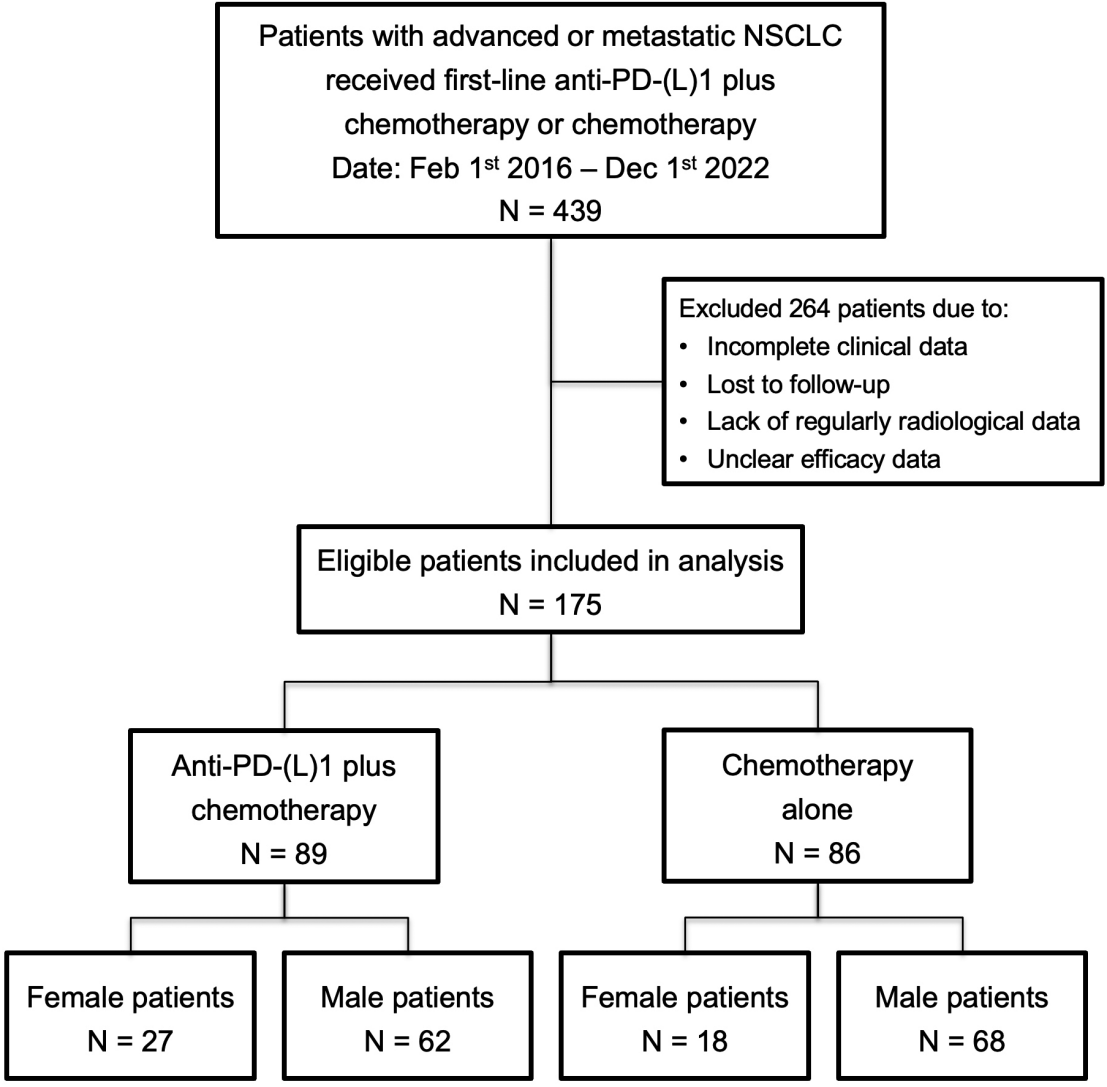
Flowchart of patients' cohort.

A total of 439 patients with untreated advanced or metastatic NSCLC treated with PD‐1 blockade combined with chemotherapy or chemotherapy alone were identified for eligibility in this study (Figure 1). Of them, 264 patients with incomplete clinical data, lack of regularly radiological data and/or unclear efficacy data were excluded. Finally, 175 of them were eligible (Figure 1). Of the 175 patients, 89 received first‐line PD‐1 blockade plus chemotherapy and 86 received first‐line chemotherapy. The clinicopathological features of the study population are listed in Table 1; Table S1. Of them, 45 were women (25.7%) and 130 were men (74.3%), with a mean age of 63 years. 56.6% of patients were never smokers or smoking history <400 cigarette‐years (*n* = 99) and 76.0% of them had an ECOG PS of 1 (*n* = 133). 134 (76.7%) patients had positive PD‐L1 expression. Patients treated with first‐line PD‐1 blockade in combination with chemotherapy had significantly prolonged PFS (11.2 vs. 6.5 months; HR = 0.52, 95% CI, 0.34–0.68) and OS (28.9 vs. 21.1 months; HR = 0.73, 95% CI, 0.50–1.04) than those received first‐line chemotherapy (Figure S1).

**TABLE 1:** Baseline characteristics of included patients.

	Anti‐PD‐(L)1 plus chemotherapy			Chemotherapy alone		
	Female (*n* = 27)	Male (*n* = 62)	*p* value	Female (*n* = 18)	Male (*n* = 68)	*p* value
Age						
≥65 years	2 (7.4%)	10 (16.1%)	0.441	6 (33.3%)	22 (32.4%)	0.937
<65 years	25 (92.6%)	52 (83.9%)		12 (66.7%)	46 (67.6%)	
Smoking history						
≥400 cigarette‐years	3 (11.1%)	60 (96.8%)	<0.001	1 (5.6%)	56 (82.4%)	<0.001
<400 cigarette‐years or never	24 (88.9%)	2 (3.2%)		17 (94.4%)	12 (17.6%)	
ECOG performance status						
0	7 (25.9%)	18 (29.0%)	0.764	5 (27.8%)	12 (17.6%)	0.531
1	20 (74.1%)	44 (71.0%)		13 (72.2%)	56 (82.4%)	
Disease stage						
IIIB/IIIC	3 (11.1%)	12 (19.4%)	0.518	2 (11.1%)	11 (16.2%)	0.870
IV	24 (88.9%)	50 (80.6%)		16 (88.9%)	57 (83.8%)	
Pathological type						
Adenocarcinoma	26 (96.3%)	59 (95.2%)	0.750	17 (94.4%)	66 (97.1%)	0.853
Squamous cell carcinoma	1 (3.7%)	3 (4.8%)		1 (5.6%)	2 (2.9%)	
No. of distant metastases						
≤3	13 (48.1%)	32 (51.6%)	0.764	10 (55.6%)	31 (45.6%)	0.452
>3	14 (51.9%)	30 (48.4%)		8 (44.4%)	37 (54.4%)	
Brain metastases at enrollment						
Yes	1 (3.7%)	1 (1.6%)	0.868	0 (0.0%)	3 (4.4%)	0.853
No	26 (96.3%)	61 (98.4%)		18 (100%)	65 (95.6%)	
PD‐L1 tumor proportion score						
<1%	4 (14.8%)	16 (25.8%)	0.387	6 (33.3%)	15 (22.1%)	0.322
≥1%	23 (85.2%)	46 (74.2%)		12 (66.7%)	53 (77.9%)	
≥50%	5 (18.5%)	7 (11.3%)		1 (5.6%)	13 (19.1%)	
Anti‐PD‐1/PD‐L1 antibodies						
Pembrolizumab	6 (22.2%)	11 (17.7%)	0.621	‐	‐	
Camrelizumab	18 (66.7%)	45 (72.6%)		‐	‐	‐
Tislelizumab	3 (11.1%)	6 (9.7)		‐	‐	
Chemotherapy						
Carboplatin plus pemetrexed	26 (96.3%)	59 (95.2%)	0.750	17 (94.4%)	66 (97.1%)	
Carboplatin plus paclitaxel	1 (3.7%)	3 (4.8%)		1 (5.6%)	2 (2.9%)	0.853

Abbreviations: ECOG, Eastern Cooperative Oncology Group performance status; PD‐(L)1, programmed cell death (ligand) 1.

### 3.2 Comparison of baseline parameters between female and male patients

Baseline features, including age, ECOG PS, number of distant metastases, brain metastasis, disease stage, pathological type, and PD‐L1 expression level were similar between male and female patients (Table 1; Table S2). However, males had a much higher rate of smoker than females (17.6% vs. 5.6%; *p* < 0.001) which was consistent with the public data of Chinese NSCLC. For all included patients, female patients had a markedly longer PFS (12.6 vs. 7.2 months; HR = 0.56, 95% CI, 0.40–0.80) and OS (35.3 vs. 21.1 months; HR = 0.59, 95% CI, 0.40–0.87) than male patients (Figure S2). In 89 patients received PD‐1 blockade plus chemotherapy, 27 of them were female and 62 were male. In 86 patients treated with chemotherapy, 18 of them were female and 68 were male. Similarly, age, brain metastasis, disease stage, ECOG PS, and PD‐L1 expression level were also similar between male and female patients in both groups, but males had a much higher rate of smoker than females (Table 1).

### 3.3 Impact of sex on the efficacy of first‐line setting

We firstly addressed the impact of sex on efficacy of first‐line PD‐1 blockade in combination with chemotherapy or chemotherapy alone. As shown in Table 2, female patients treated with first‐line PD‐1 blockade plus chemotherapy had a dramatically better ORR than male patients (85.2% vs. 53.2%; *p* = 0.009). Female patients also had a higher DCR than male patients (100% vs. 85.5%; *p* = 0.088) but it did not reach the statistical significance. In chemotherapy group, female patients had analogous ORR (33.3% vs. 41.2%; *p* = 0.545) and DCR (94.4% vs. 79.4%; *p* = 0.252) to male patients.

**TABLE 2:** Summary of response rate in female versus male patients.

	Anti‐PD‐(L)1 plus chemotherapy			Chemotherapy alone		
	Female (*n* = 27)	Male (*n* = 62)	*p* value	Female (*n* = 18)	Male (*n* = 68)	*p* value
Complete response	0 (0.0%)	1 (1.6%)		0 (0.0%)	0 (0.0%)	
Partial response	23 (85.2%)	32 (51.6%)		6 (33.3%)	28 (41.2%)	
Stable disease	4 (14.8%)	20 (32.3%)		11 (61.1%)	26 (38.2%)	
Progression disease	0 (0.0%)	9 (14.5%)		1 (5.6%)	14 (20.6%)	
Objective response rate	23 (85.2%)	33 (53.2%)	0.009	6 (33.3%)	28 (41.2%)	0.545
Disease control rate	27 (100.0%)	53 (85.5%)	0.088	17 (94.4%)	54 (79.4%)	0.252

**FIGURE 2:**
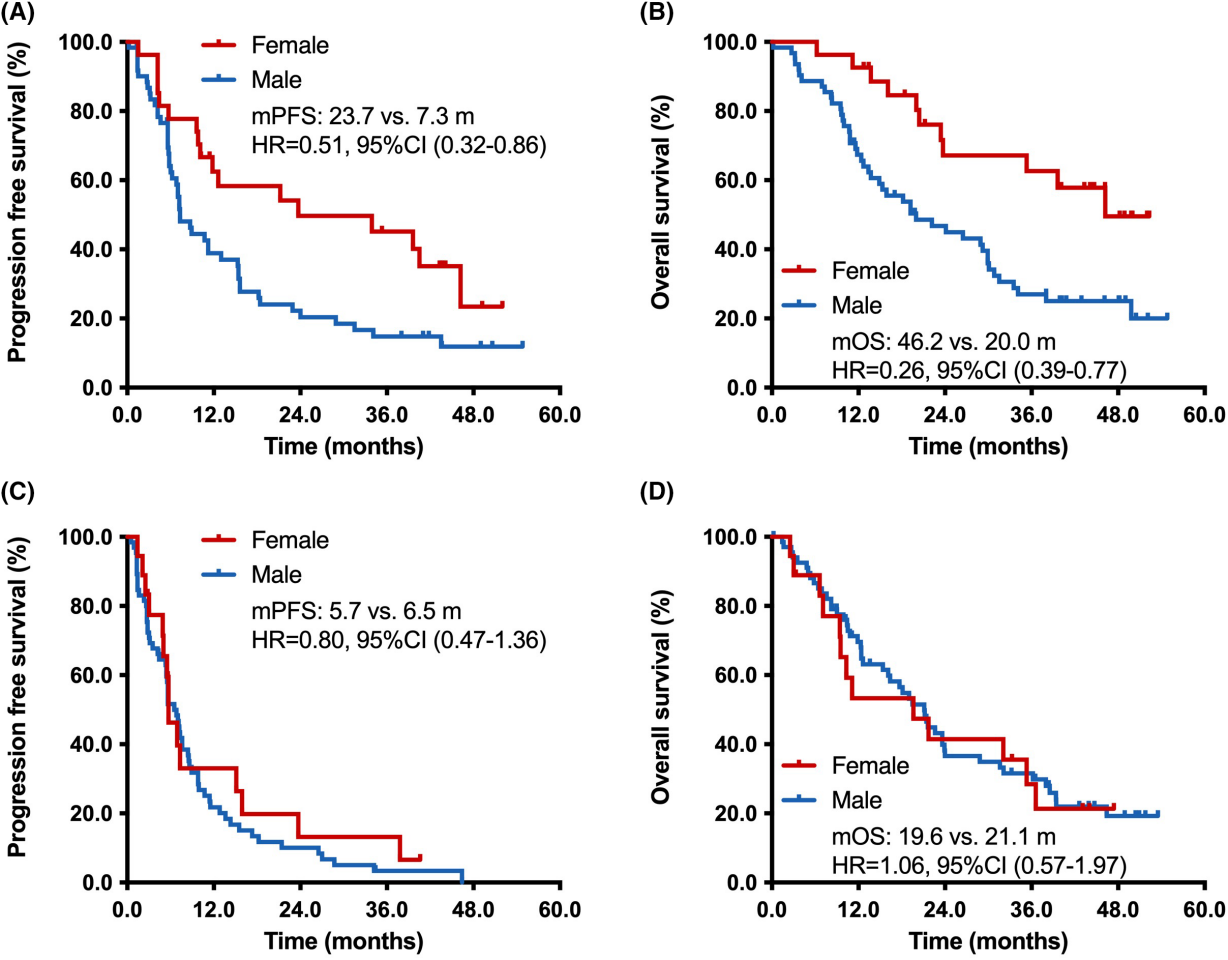
The impact of sex on the efficacy of first‐line PD‐1 blockade plus chemotherapy or chemotherapy in patients with NSCLC. (A). Comparison of PFS between female and male patients received first‐line PD‐1 blockade plus chemotherapy; (B) Comparison of OS between female and male patients received first‐line PD‐1 blockade plus chemotherapy; (C) Comparison of PFS between female and male patients received first‐line chemotherapy; (D) Comparison of OS between female and male patients received first‐line chemotherapy. CI, confidence interval; HR, hazard ratio; mPFS, median progression‐free survival; mOS, median overall survival.

Next, we tested the effect of sex on PFS and OS of first‐line setting. The results showed a significantly longer PFS for female patients compared with male patients in first‐line PD‐1 blockade plus chemotherapy group (23.7 vs. 7.3 months; HR = 0.51, 95% CI, 0.32–0.86; *p* = 0.013; Figure 2A). Importantly, female patients treated with first‐line PD‐1 blockade combined with chemotherapy had an obviously superior OS than male patients (46.2 vs. 20.0 months; HR = 0.26, 95% CI, 0.39–0.77; *p* = 0.004; Figure 2B). In chemotherapy group, female patients had similar PFS (5.7 vs. 6.5 months; HR = 0.80, 95% CI, 0.47–1.36; *p* = 0.421; Figure 2C) and OS (19.6 vs. 21.1 months; HR = 1.06, 95% CI, 0.57–1.97; *p* = 0.862; Figure 2D) to male patients.

### 3.4 Multivariate analyses

**FIGURE 3:**
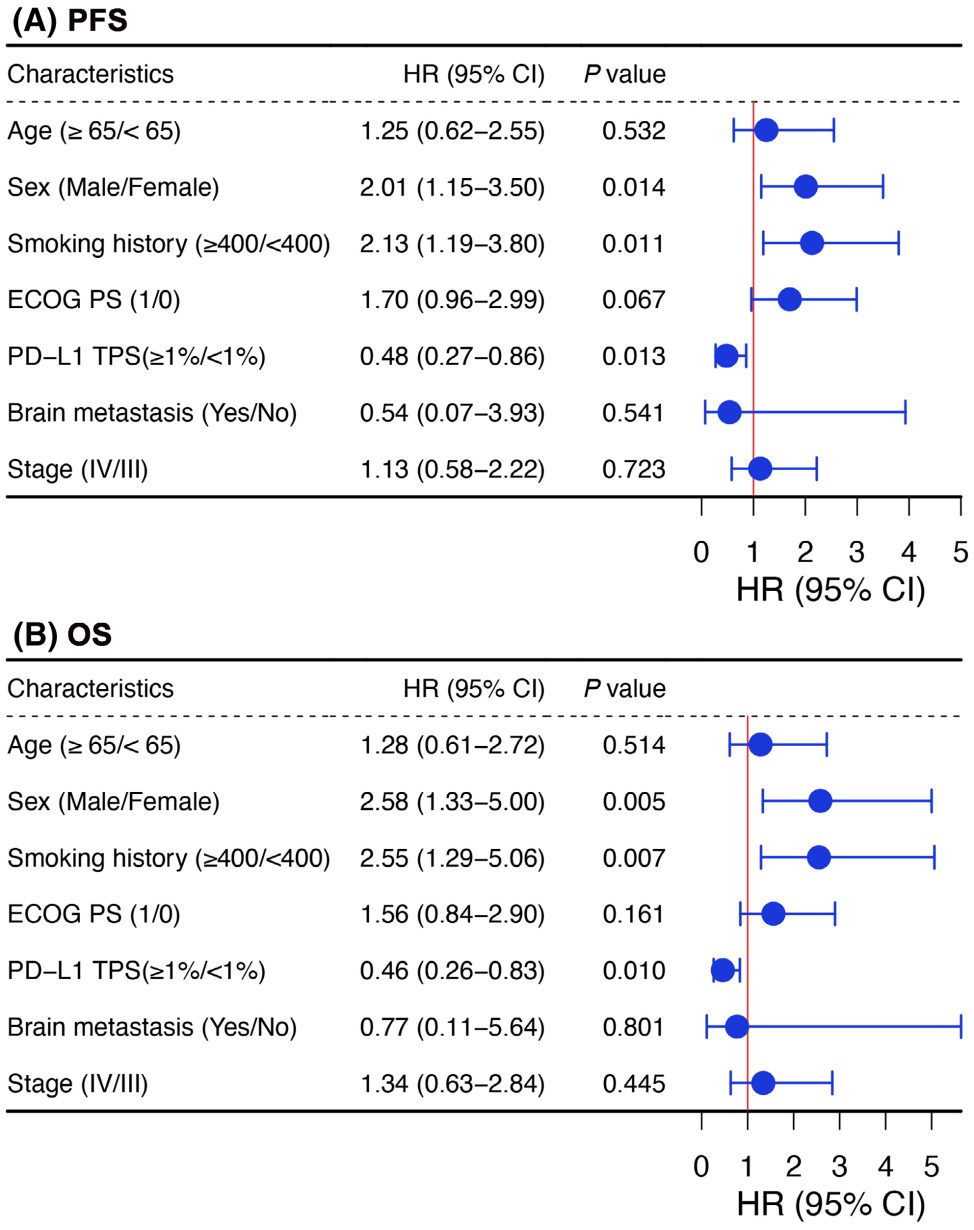
Forrest plot of multivariate analyses in patients received first‐line PD‐1 blockade plus chemotherapy. (A) Forrest plot of multivariate analyses on PFS; (B) forest plot of multivariate analyses on OS. CI, confidence interval; HR, hazard ratio; TPS, tumor proportional score.

Considering the limited number of clinicopathological features, we performed the multivariate analyses to include all the listed baseline parameters to identify the independent predictive and/or prognostic factors. We observed that male sex was independently associated with the markedly shorter PFS than female sex (HR = 2.01, 95% CI, 1.15–3.50; *p* = 0.014; Figure 3A). In addition, smoking history and positive PD‐L1 expression level were also independently correlated with PFS. As for OS, multivariate analyses showed that male sex was also independently associated with the shorter OS than female sex (HR = 2.58, 95% CI, 1.33–5.00; *p* = 0.005; Figure 3B). Analogously, smoking history and positive PD‐L1 expression level were also independently correlated with OS. However, in patients treated with chemotherapy, sex was not correlated with both PFS and OS (Figure S3).

### 3.5 Tumor mutation burden and neoantigen heterogeneity

**FIGURE 4:**
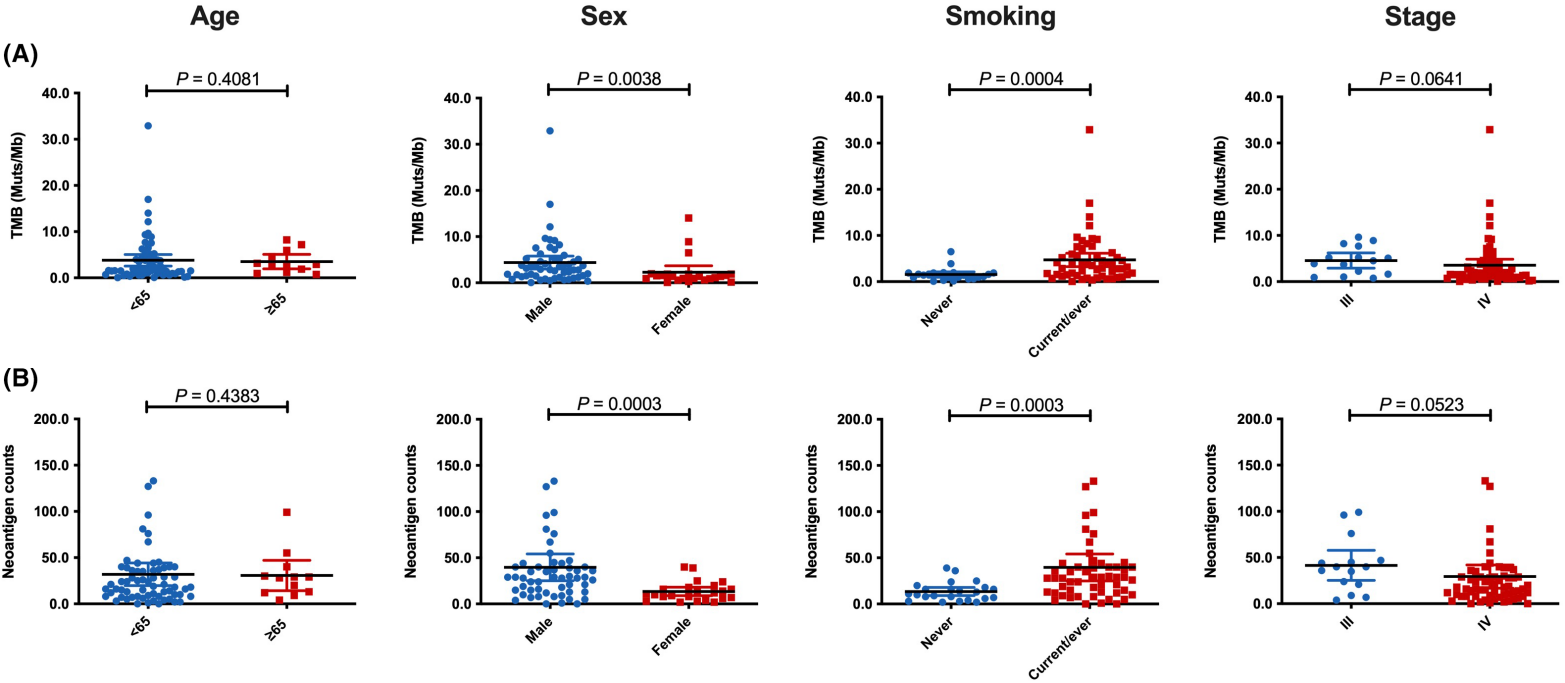
Comparisons of tumor mutation and neoantigen burden between patients with distinct clinical parameters. (A) Comparisons of tumor mutation burden between patients with distinct clinical parameters including age, sex, smoking history, and stage. (B) Comparisons of neoantigen burden between patients with distinct clinical parameters including age, sex, smoking history, and stage.

To compare the tumor mutation burden and neoantigen heterogeneity between patients with distinct clinical parameters including age, sex, smoking, and stage, we performed the whole‐exome sequencing to the pretreatment tumor tissue samples and analyzed the tumor mutation and neoantigen burden. Seventy‐seven patients in PD‐1 blockade combined with chemotherapy group had the high‐quality tissue samples. As shown in Figure 4, we observed that age and stage had no impact on the tumor mutation and neoantigen burden. Ever/current smokers had markedly higher tumor mutation and neoantigen burden than never smokers. Notably, female patients had significantly lower tumor mutation and neoantigen burden than male patients (Figure 4).

### 3.6 Tumor immune microenvironmental features analysis

**FIGURE 5:**
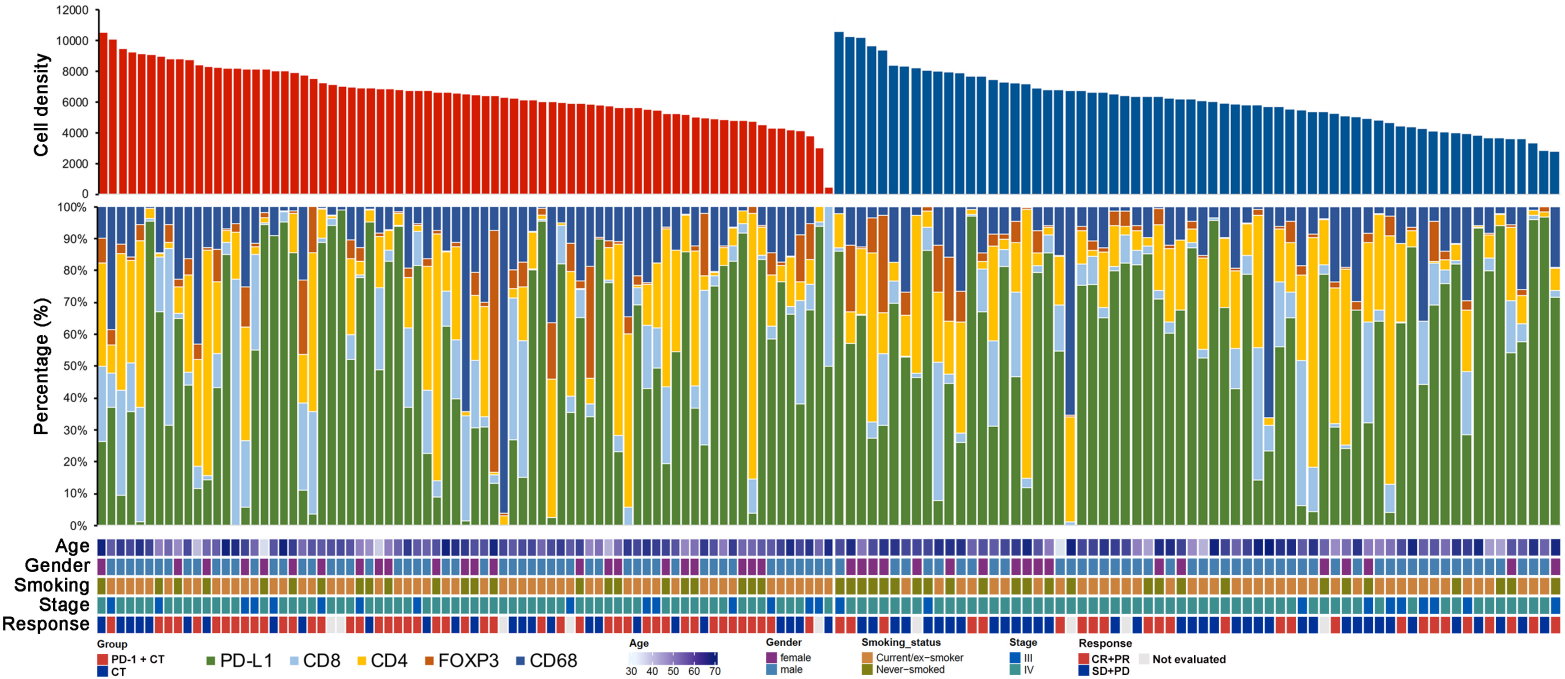
Expression landscape of five tumor immune microenvironmental markers in 144 patients with advanced NSCLC. Upper panel: The number of identified stained cell. Middle panel: expression level of five tumor immune microenvironmental markers. Bottom panel: major clinicopathological characteristics. Columns represent samples.

**FIGURE 6:**
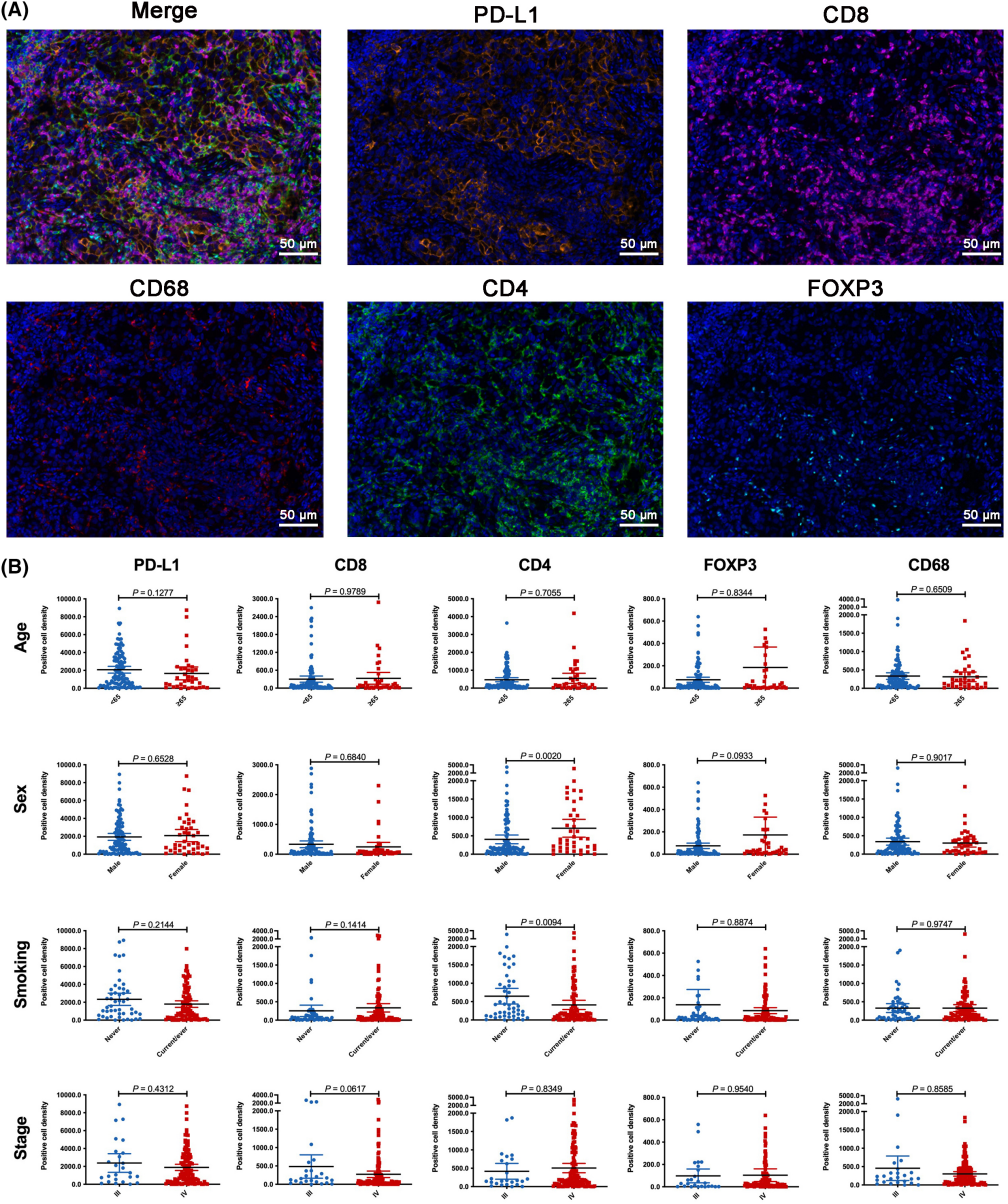
Comparisons of tumor immune microenvironmental markers between patients with distinct clinical parameters. (A) Representative mIF images of PD‐L1, CD8, CD68, CD4, FOXP3 expression. (B) Comparisons of each tumor immune microenvironmental markers expression level, including PD‐L1, CD8, CD68, CD4, and FOXP3, between patients with distinct clinical parameters, and comparisons of the expression level of five rational combinations of these markers, including CD8/PD‐L1, CD68/PD‐L1, CD4/FOXP3, CD8/CD68/PD‐L1, and CD4/FOXP3/PD‐L1, between patients with distinct clinical parameters.

To unravel the potential biological explanations on the sex‐base heterogeneity in treatment response, we then surveyed the expression level of CD8, CD4, CD68, FOXP3, and PD‐L1 between male and female patients. 144 (82.3%) of them had the adequate and high‐quality tissue samples for mIF analysis. The expression level of each marker was recorded as the percent of stained cells and positive cell density score. We found that expression patterns of tumor immune microenvironmental related markers varied broadly, ranging from “immune‐cold” tumors with few or no marker expression to strongly expressed tumors (Figure 5). We also listed the representative images of five tumor immune microenvironmental related markers expression in Figure 6A. As shown in Figure 6B, age, smoking history, and stage showed very limited effect on the expression level differences of tumor immune microenvironmental related markers, whereas female patients had significantly both higher CD4 positive cell density score (*p* = 0.002) than male patients. When we used the percent of stained cells, we observed the similar results (Figure S4). Other markers, including PD‐L1, CD68, FOXP3, and CD8, showed analogous expression level between female and male patients.

**FIGURE 7:**
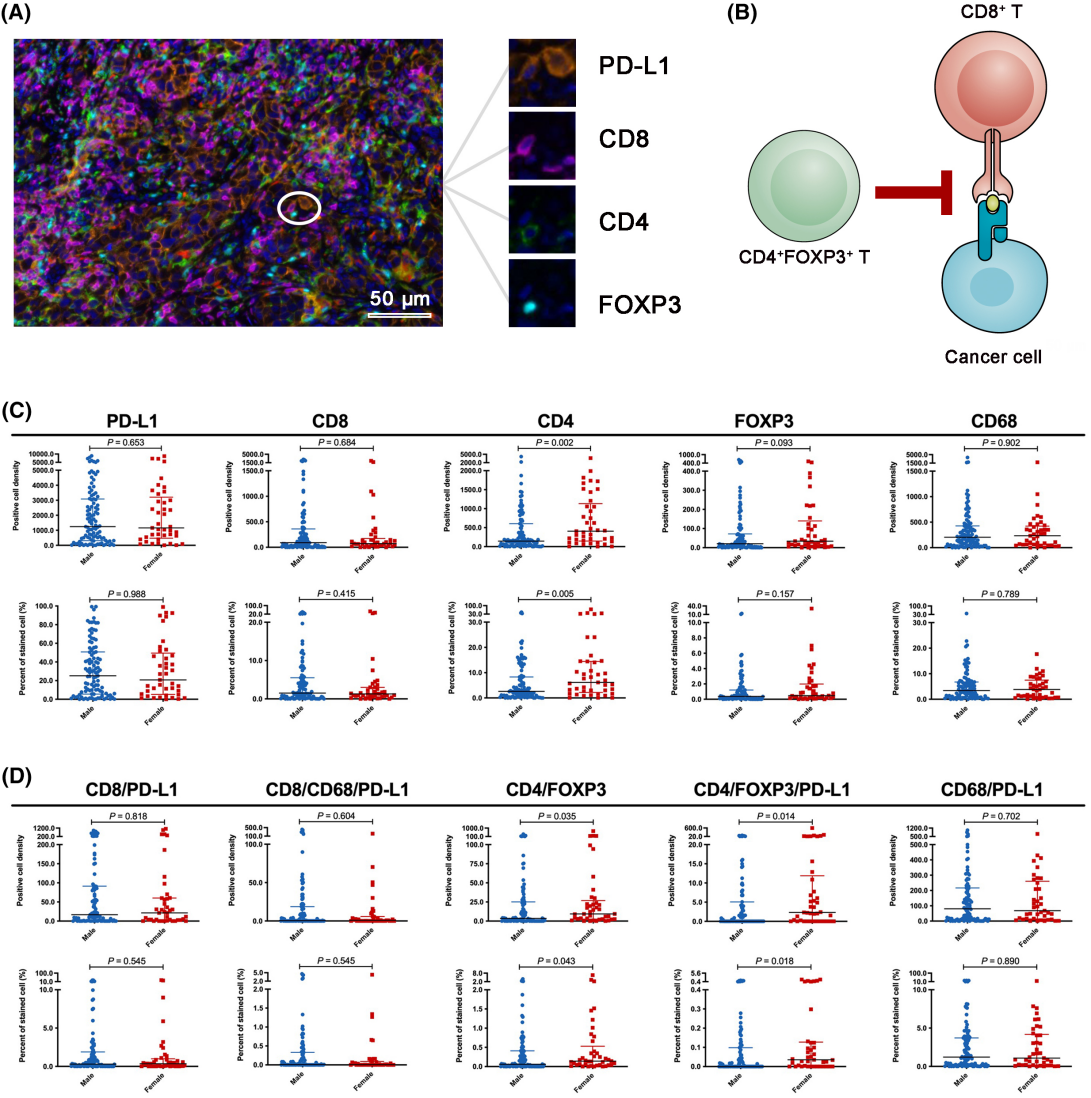
Comparisons of tumor immune microenvironmental markers and their combinations between male and female patients. (A) Representative mIF images of marker co‐expression. (B). CD4/FOXP3 Tregs inhibit CD8^+^ T cells cytotoxicity. (C) Comparisons of each tumor immune microenvironmental markers expression level, including PD‐L1, CD8, CD68, CD4, and FOXP3, between male and female patients. (D) Comparisons of the expression level of five rational combinations of these markers, including CD8/PD‐L1, CD68/PD‐L1, CD4/FOXP3, CD8/CD68/PD‐L1, and CD4/FOXP3/PD‐L1, between male and female patients.

Considering distinct marker combinations could represent the relative abundance of specific immune cells and immune response process (Figure 7A,B), we then tested the correlations between their biologically rational combinations and sex. We observed that female patients had dramatically higher CD4/FOXP3 and CD4/FOXP3/PD‐L1 co‐expression level than male patients (Figure 7C,D), whereas CD68/PD‐L1, CD8/PD‐L1, and CD8/CD68/PD‐L1 showed analogous co‐expression level between female and male patients (Figure 7C,D).

## 4 DISCUSSION

Accumulating evidence suggest that sex could affect the adaptive antitumor immunity. Thus, in the current study, we evaluated the impact of sex on the efficacy of first‐line PD‐1 blockade in combination with chemotherapy or chemotherapy alone in patients with advanced or metastatic NSCLC. We found that female patients treated with first‐line PD‐1 blockade combined with chemotherapy had dramatically better treatment outcomes than male patients, while treatment outcomes showed no difference in chemotherapy group. Moreover, multivariate analyses showed that sex was the independent prognostic factor for patients received PD‐1 blockade in combination with chemotherapy, instead of chemotherapy. Furthermore, we observed that pretreatment tumor tissues from female patients had markedly lower tumor mutation and neoantigen burden, but significantly higher CD4, CD4/FOXP3, and CD4/FOXP3/PD‐L1 expression level than male patients. Collectively, these findings indicate that female patients with untreated advanced NSCLC would derive a larger benefit from PD‐1 blockade in combination with chemotherapy than male patients. The differences of neoantigen burden and tumor immune microenvironmental features between female and male patients may be the potential biological explanation and warrant future detailed investigations.

Given the existence of sex‐related dimorphism in immune functions and responses, several studies began to evaluate the treatment response difference of ICI between female and male patients with advanced cancers. In 2018, Fabio et al. conducted a meta‐analysis to evaluate the heterogeneity of ICI efficacy between women and men with advanced or metastatic cancers. They identified 20 high‐quality clinical trials with 11,351 patients and observed that men had dramatically higher survival benefit than women.^20^ Considering the various cancer types, distinct study design, population, and drugs, they cannot give a definite conclusion in a certain cancer type. In 2019, Fabio et al. performed an update meta‐analysis to investigate the sex‐related response heterogeneity to cancer immunotherapy and found that females with advanced or metastatic lung cancer could have a dramatically larger benefit from PD‐1 blockade plus chemotherapy.^21^ More recently, several update meta‐analyses also reported that female patients had a greater treatment outcomes improvement than male patients.^22^, ^23^, ^24^ Consistently, this study analyzed 175 patients with untreated advanced or metastatic NSCLC received PD‐1 blockade plus chemotherapy or chemotherapy and also observed that female patients derived a larger benefit from first‐line PD‐1 blockade in combination with chemotherapy, but not chemotherapy, than male patients. Taken together, these findings suggest that female patients with untreated advanced or metastatic NSCLC could derive a larger benefit from PD‐1 blockade plus chemotherapy. Future investigations should focus on exploring novel and rational immunotherapeutic combination strategies to improve clinical outcomes of male patients.

To unravel the underlying mechanism of the impact of sex on the antitumor immune response, several recent publications focus on this research area. Hyunwoo et al. reported that androgen could promote the CD8^+^ T cell dysfunction via activating intrinsic androgen receptor (AR).^18^ Blockade of the androgen‐AR axis could shape the tumor microenvironment to support effector T cell differentiation and potentiate the efficacy of PD‐1 blockade. Yand and colleagues further demonstrated that androgen‐AR signaling could promote the stem cell‐like CD8^+^ T cells transition to terminally exhausted CD8^+^ T cells in males, resulting in sex‐related antitumor immunity. Ablation of AR signaling could maintain CD8^+^ T cell stemness.^17^ Guan et al. also found that blockade of AR signaling in CD8^+^ T cells could prevent T cell exhaustion and improved response to PD‐1 blockade via increased IFNγ expression.^19^ Collectively, these results suggest that gonadal androgen plays a pivotal role in treatment response via regulating CD8^+^ T cell–dependent antitumor immunity, shedding insights into the future development of sex‐based therapeutic strategies.

Having noticed the significant impact of sex on the antitumor immunity and efficacy of immunotherapy, we also conducted the tumor immune microenvironmental features analysis to compare the neoantigen burden and expression level of several important immune markers between female and male patients. We firstly evaluated the heterogeneity of neoantigen burden between male and females in PD‐1 blockade in combination with chemotherapy group. The results showed that female patients had markedly lower tumor mutation and neoantigen burden than males. Then we analyzed the pretreatment tumor tissues from 144 patients and found that female patients had a markedly higher CD4 expression level. Further analysis revealed that CD4/FOXP3 and CD4/FOXP3/PD‐L1 co‐expression level was much higher in female than in male patients. These findings suggest that CD4^+^ T cell could also play a role in sex‐related difference on the response to PD‐1 blockade combined with chemotherapy. Nevertheless, the reason why female patients had higher expression level of CD4, CD4/FOXP3, and CD4/FOXP3/PD‐L1 than male patients remains largely unknown. Considering CD4^+^ T cell could help CD8^+^ T cell‐mediate cytotoxicity and directly kill the cancer cells,^28^, ^29^, ^30^ future research are warranted to clarify the role of CD4^+^ T cell in sex‐based heterogeneity of treatment response.

The present study had several limitations. First, although the initial cohort is large, the final analysis cohort was small and the selection bias was inevitable due to retrospective nature. Hence, the current findings should be cautiously interpreted and still need future validation with large sample size. Second, given the small sample size, we cannot divide the final analysis cohort into a test and validation cohort, and perform the subgroup analysis according to the smoking history. The lack of validation in a separate dataset could limit the immediately clinical applicability of the present findings. The impact of smoking history on sex‐related difference of treatment outcomes need further investigations. Third, patients received first‐line PD‐1 blockade in combination with chemotherapy were mainly identified from October 2019 to December 2022. Patients received chemotherapy were mainly identified from February 2016 to December 2018. The different years of patients' identification would lead to the outcome assessment bias. Fourth, we did not put all the potential prognostic/predictive factors (e.g., histology, liver or bone metastasis, number of sites of metastases, history of previous therapy for nonmetastatic disease) into the multivariate analysis. Thus, these findings should be interpreted with caution. Last but not least, the current study only included Chinese NSCLC patients. Given the different genetics and immunophenotype of patients from distinct ethnicities, it should be cautious to apply the current findings to other races.

In summary, the present findings revealed that female patients with untreated advanced or metastatic NSCLC would derive a larger benefit from PD‐1 blockade in combination with chemotherapy than males. The differences of neoantigen burden and tumor immune microenvironmental features, especially CD4^+^ T cell composition and function, between female and males may be the potential biological explanation and worthy of further investigation.

## AUTHOR CONTRIBUTIONS

**Xiaofeng Yu:** Conceptualization (equal); data curation (equal); formal analysis (equal); funding acquisition (equal); investigation (equal); methodology (equal); supervision (equal); validation (equal); visualization (equal); writing – original draft (equal). **Zhaolei You:** Conceptualization (equal); project administration (equal); resources (equal); software (equal); supervision (equal); validation (equal); visualization (equal); writing – original draft (equal). **Ying Liu:** Formal analysis (equal); funding acquisition (equal); investigation (equal); methodology (equal); project administration (equal). **Jian Fang:** Methodology (equal); project administration (equal); resources (equal); software (equal); supervision (equal); validation (equal). **Qi Zhao:** Investigation (equal); methodology (equal); project administration (equal); resources (equal); software (equal); supervision (equal). **Zhihong Sun:** Data curation (equal); formal analysis (equal); resources (equal); software (equal); supervision (equal); validation (equal); visualization (equal). **Yingjian Song:** Investigation (equal); project administration (equal); supervision (equal); validation (equal); visualization (equal). **Jie Liu:** Conceptualization (equal); data curation (equal); formal analysis (equal); investigation (equal). **Chengming Sun:** Conceptualization (lead); data curation (lead); funding acquisition (lead); investigation (lead); writing – original draft (lead); writing – review and editing (lead).

## FUNDING INFORMATION

This work was partly supported by grants from the Natural Science Foundation of Shandong Province (ZR202110270023) and the Science and Technology Plan of Yantai city (2022YD037).

## CONFLICT OF INTEREST STATEMENT

The authors declared no potential conflicts of interest.

## ETHICS STATEMENT

This study was approved by Institutional Review Board of Yantai Yuhuangding Hospital. Written informed consent for participation was waived for this retrospective analysis in accordance with the national legislation and the institutional requirements. The study protocol was conducted according to the Declaration of Helsinki, and local laws and regulations of China.

## Supporting information


Data S1.

## Data Availability

The raw data supporting the conclusions of this work were summarized in Table S4 and will be available by the corresponding authors on reasonable request.
