# Supplementary material for: Sex‐based immune microenvironmental feature heterogeneity in response to PD‐1 blockade in combination with chemotherapy for patients with untreated advanced non‐small‐cell lung cancer
Source: Cancer Med. 2024 Jun 20;13(12):e7423. doi: 10.1002/cam4.7423 (PMC11188036; doi:10.1002/cam4.7423)
Supplement: Supplementary file 1 — Data S1. [file CAM4-13-e7423-s001.docx]

**Sex-based immune microenvironmental feature heterogeneity in response to PD-1 blockade in combination with chemotherapy for patients with untreated advanced non-small-cell lung cancer**

**Supplemental Materials**

**Online methods…………………………………………………………………………..2**

**Supplemental figure S1…………………………………………………………………7**

**Supplemental figure S2…………………………………………………………………8**

**Supplemental figure S3…………………………………………………………………9**

**Supplemental figure S4…………………………………………………………………10**

**Supplemental table S1………………………………………………………………..…11**

**Supplemental table S2………………………………………………………………..…12**

**Supplemental table S3………………………………………………………………..…13**

**Supplemental table S4………………………………………………………………..…14**

**References…………………………………………………………………………………20**

**Online methods**

**Library preparation**

30 to 300 ng of genomic DNA was sheared to length of approximately 200 base pair (bp) by Covaris LE220 and library preparations were performed with KAPA Hyper Prep Kit (KAPA Biosystems, Wilmington, MA, USA). Libraries were quantified with quantitative PCR using the KAPA Library Quantification kit (KAPA Biosystems), and the size was determined using the Bioanalyzer 2100 (Agilent Technologies, Santa Clara, CA, USA). Sequencing was performed on the Illumina HiSeq4000 platform using PE150 sequencing chemistry (Illumina, San Diego, CA, USA).

**Data processing and variants calling**

Base calling was performed on bcl2fastq V.2.16.0.10(1) to generate sequence reads in the FASTQ format. After removing low quality reads by Trimmomatic (v0.36)(2), clean reads were aligned to the human reference genome (hg19, NCBI Build 37.5) with the Burrows-Wheeler Aligner (BWA, version 0.7.17). Then Picard toolkit (version 2.23.0)(3) was used to convert SAM files to compressed BAM files, which were then sorted according to chromosome coordinates. The Genome Analysis Toolkit (GATK)(4) was used for realignment. Single nucleotide variants (SNVs) and small insertions/deletions (InDels) were called via MuTect2(5) with tumor-normal mode. To avoid false-positive results, SNVs and InDels that appeared on the blacklist (including sequence-specific errors, repeat regions, segmental duplications and lowly mappable regions recorded in ENCODE) were removed. After annotation by ANNOVAR(6), we filtered out variants either in introns or synonymous mutations. Somatic mutations with following criterions were used for analysis: (i) the sequencing depth was more than 100×; (ii) The variant allele frequency (VAF) threshold of SNV was 4% and that of InDels was 5%. Furthermore, variants with minor allele frequency ≥1% in the Exome Aggregation Consortium (ExAC) and Genome Aggregation Database (gnomAD) were removed. The mutations that were not recorded in the COSMIC database were filtered out. Somatic copy number variations (CNVs) were identified using the CNVkit (v0.9.5).

**TMB calculation**

As previously described(7), TMB was calculated by integrating the total number of somatic, base substitutions, coding, and InDel mutations per megabases of the genome examined. We firstly quantified the number of somatic nonsynonymous SNVs. Then the value was extrapolated to the whole exome using a validated algorithm(8). Only the regions with sequencing depth >100× after deduplication were used for TMB calculation. Germline genomic alterations in the Single Nucleotide Polymorphism database or occurring with ≥2 counts in the ExAC database were not recorded.

**Neoantigen prediction**

Similar to the previous study(7), we calculated the neoantigen burden by using the following algorithm. First, we called the HLA-I alleles from the matched normal exome sequencing data using HLA-HD (v1.2.0.1)(9). Neoepitope presentation was then predicted for tumor-specific peptides of length 9-11 using the eluted-ligand mode of NetMHCpan-4.0(10). The following criteria were used to select the potential neoantigens for subsequent analysis: (i) derived from tumor-specific genomic alterations (including missense, inframe indels, frameshift, and fusions); (ii) high predicted affinity to HLA-I alleles [half maximal inhibitory concentration (IC50) < 500 nmol/L] with k-mer of 9-11 length; (iii) fold change >10 comparing to wild-type binding affinity. IEDB-recommended model was conducted to predict the HLA binding affinity using all variant-containing 9-11 mer for HLA-A/B/C binding estimations. HLA typing for patients was performed in silico using HLA-ATHLATES(11) per the recommended algorithm.

**mIF staining**

Manual mIF staining was performed in 4-μm sequential histologic tumor sections obtained from FFPE tumor blocks using the Opal 7-Color fIHC Kit (Akoya Biosciences, Marlborough, MA, USA) based on the Tyramide Signal Amplification (TSA; Biotium, Fremont, CA, USA). The stained slides were scanned by a Vectra multispectral microscope (Akoya Biosciences). The IF markers were grouped into the panel consisting of FOXP3 (dilution 1:100; Abcam, Shanghai, China), CD8 (dilution 1:400; Zsbio, Beijing, China), PD-L1 (clone SP142, dilution 1:25, Zsbio, Beijing, China), CD4 (dilution 1:100; Zsbio, Beijing, China), and CD68 (dilution 1:500; Zsbio, Beijing, China). Briefly, the slides were deparaffinized, rehydrated, and subjected to epitope retrieval by boiling in Tris-EDTA buffer (pH 9.0; Zsbio, Beijing, China) for 20 minutes at 97°C. Endogenous peroxidases were then blocked by incubation in Antibody Diluent/Block (Akoya Biosciences) for 10 minutes. Only one antigen was detected in each round, including primary antibody incubation, secondary antibody incubation, TSA visualization, followed by labeling the next antibody after epitope retrieval and protein blocking as before. In this panel, antigens were detected in the following order: FOXP3, CD8, PD-L1, CD4 and CD68. Finally, these slides were stained with 4’, 6-Diamidino-2-Phenylindole (DAPI; Selleckchem, Shanghai, China) for nuclei and mounted with anti-quenching sealing tablets. Human tonsil FFPE tissues were used with and without primary antibodies as positive and negative controls, respectively.

**mIF image acquisition**

The stained slides were scanned with a Vectra 3.0 microscope system (PerkinElmer Waltham, MA, USA) under fluorescent illumination. From each slide, Vectra automatically captured the fluorescent spectra from 420 nm to 720 nm at 20 nm intervals with the same exposure time and then combined the captured images to create a single stack image that retained the particular spectral signature of all markers. After the specimens were scanned at low magnification (×10), the typical fields in the tumor area were scanned at high resolution (×20) to capture various elements of tumor heterogeneity.

**Multispectral analysis**

Tumor multispectral images were analyzed by two experienced pathologists using the tissue segmentation tool of the InForm 3.0 software (Akoya Biosciences). Representative multispectral images were selected as training samples to build an algorithm (tissue segmentation, cell segmentation, phenotyping tool and positivity score) using the inForm software (Akoya Biosciences). All markers in the panel were analyzed one by one at a time in the same project, and all cases were analyzed with the same algorithm. After batch analysis, the performance of the algorithm was evaluated visually for all cases. The expression level was recorded as the positive cell density score and percent of stained cells. The definition of PD-L1 tumor proportion score is different from the definition of the percent of stained cells positive. PD-L1 tumor proportion score, as measured by the 22C3 assay using immunohistochemical staining, is defined as the percentage of viable tumor cells showing partial or complete membrane staining at any intensity. The percent of stained cells positive in this study, as measured by the SP142 assay using multiplex immunofluorescence staining, is defined as the positive marker expression group as that with a positive percent of stained cells ≥median level of the study cohort.


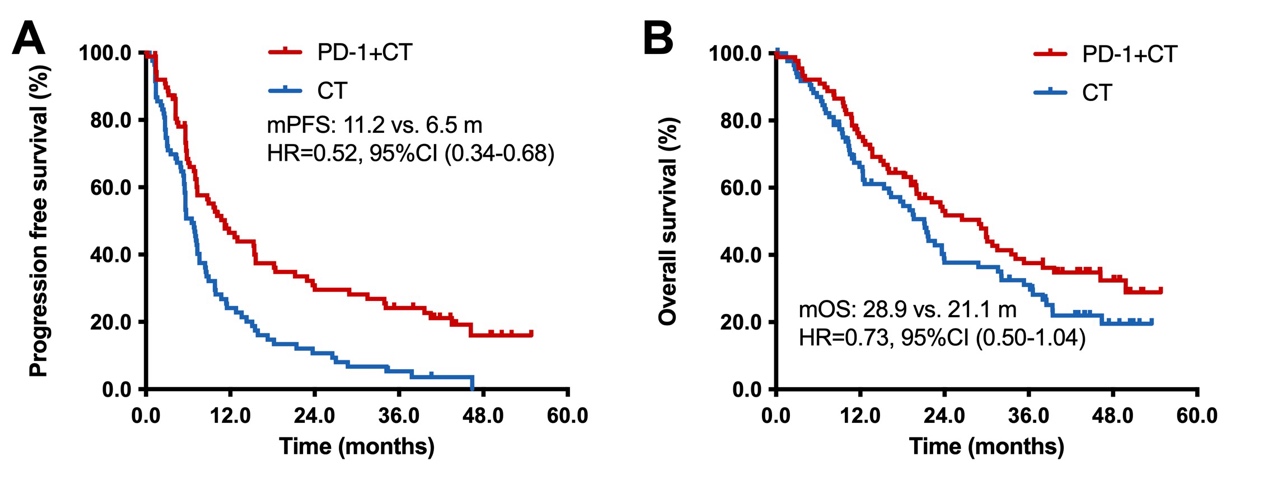


**Supplemental figure S1. Comparisons of PFS and OS in patients treated with first-line PD-1 blockade plus chemotherapy versus chemotherapy. A.** PFS comparison; **B.** OS comparison.


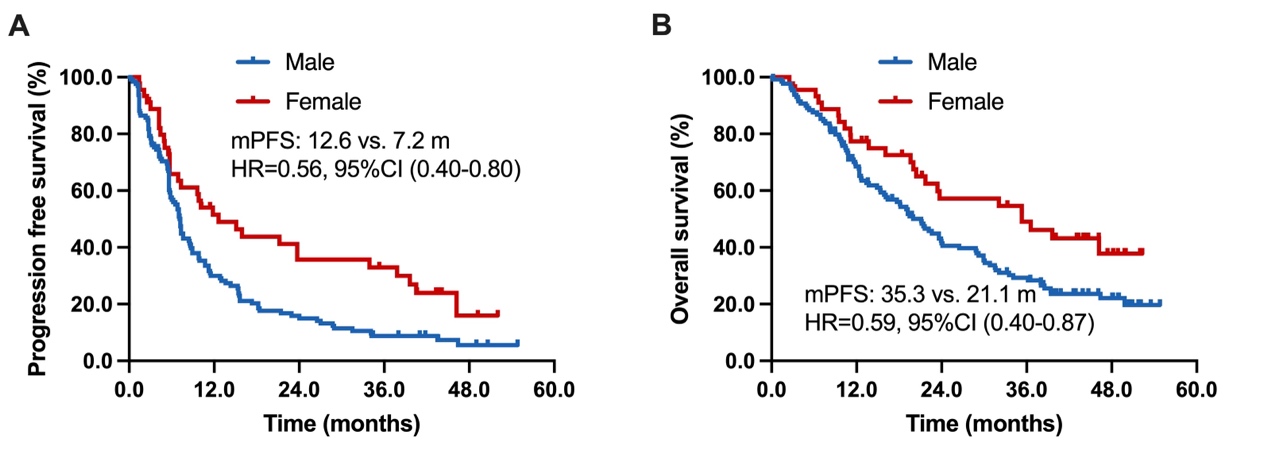


**Supplemental figure S2. The impact of sex on PFS and OS in all included patients with advanced or metastatic NSCLC. A.** The impact of sex on PFS and OS; **B.** The impact of sex on OS.


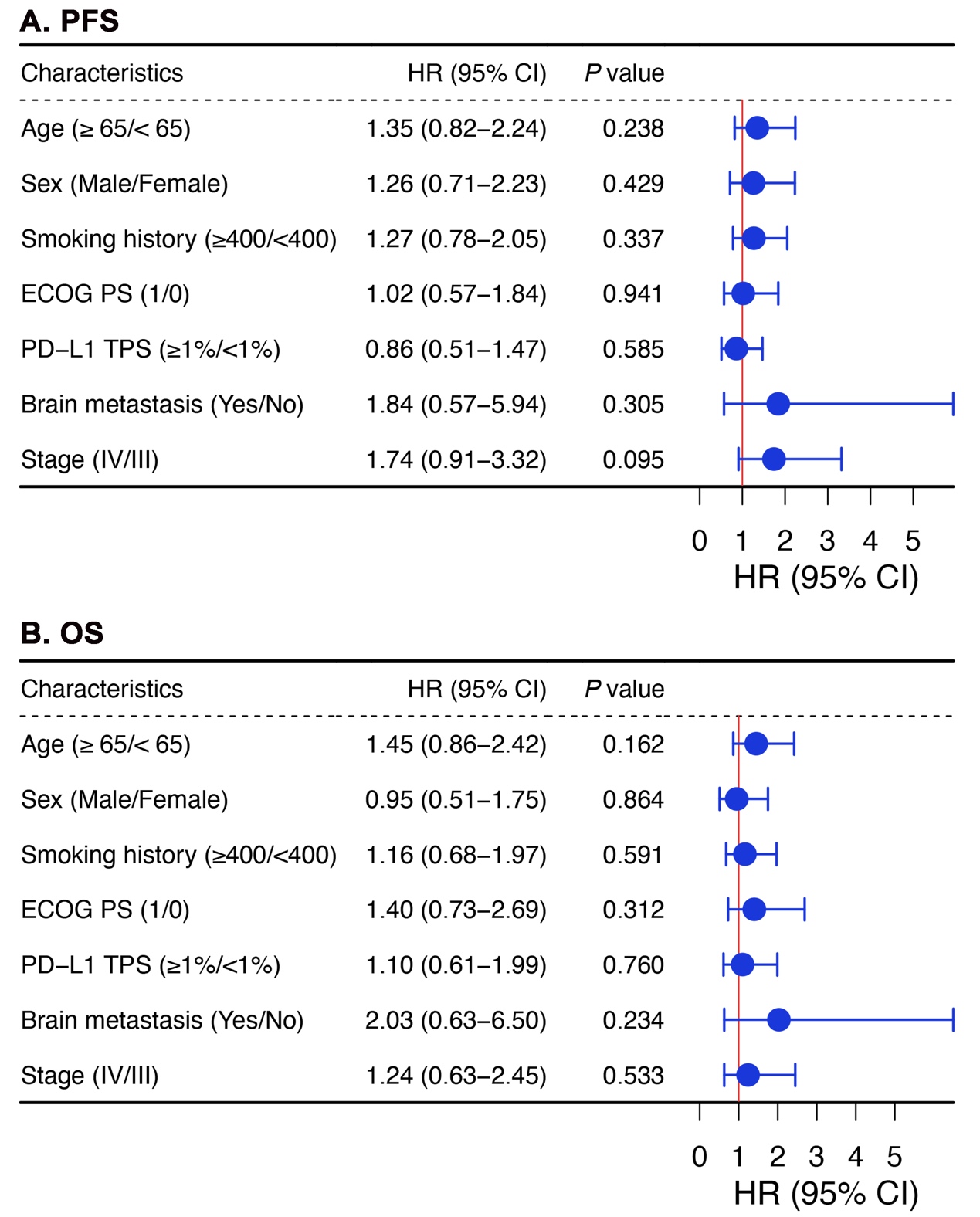


**Supplemental figure S3. Forrest plot of PFS and OS in patients treated with first-line chemotherapy. A.** Forrest plot of PFS; **B.** Forrest plot of OS.


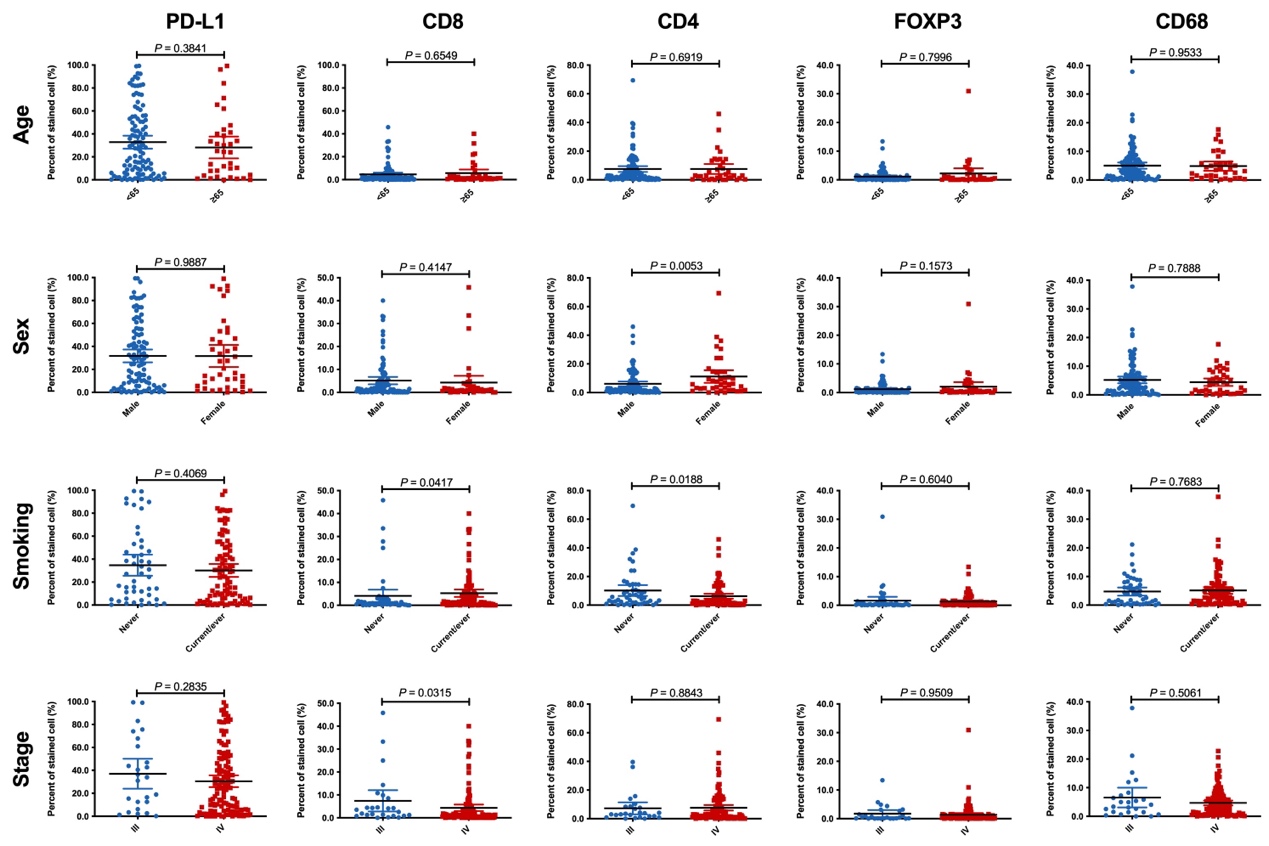


**Supplemental figure S4. Comparisons of tumor immune microenvironmental markers between patients with different clinical features.**

## Supplemental Table S1: Summary of all included patients.

|  | **Total** |  | |
| --- | --- | --- | --- |
|  | **(n=175)** |  | |
| Age |  |  | |
| ≥65 years | 40 (22.9%) |  | |
| <65 years | 135 (77.1%) |  | |
| Sex |  |  | |
| Female | 45 (25.7%) |  | |
| Male | 130 (74.3%) |  | |
| Smoking history |  |  | |
| ≥400 cigarette-years | 76 (43.4%) |  | |
| <400 cigarette-years or never | 99 (56.6%) |  | |
| ECOG performance status | | |  |
| 0 | 42 (24.0%) |  | |
| 1 | 133 (76.0%) |  | |
| Disease stage |  |  | |
| IIIB/IIIC | 28 (16.0%) |  | |
| IV | 147 (84.0%) |  | |
| Brain metastases at enrollment^*^ |  |  | |
| Yes | 5 (2.9%) |  | |
| No | 170 (97.1%) |  | |
| PD-L1 tumor proportion score | | |  |
| <1% | 41 (23.4%) |  | |
| ≥1% | 134 (76.7%) |  | |

ECOG, Eastern Cooperative Oncology Group performance status; PD-(L)1, programmed cell death (ligand) 1.

## Supplemental Table S2: Comparison of baseline characteristics between male and female patients.

|  | **Female** | **Male** | ***P* value** |
| --- | --- | --- | --- |
|  | **(n=45)** | **(n=130)** |  |
| Age |  |  |  |
| ≥65 years | 8 (33.3%) | 32 (32.4%) | 0.347 |
| <65 years | 37 (66.7%) | 98 (67.6%) |  |
| Smoking history |  |  |  |
| ≥400 cigarette-years | 4 (5.6%) | 72 (17.6%) | <0.001 |
| <400 cigarette-years or never | 41 (94.4%) | 58 (82.4%) |  |
| ECOG performance status | | |  |
| 0 | 12 (27.8%) | 30 (17.6%) | 0.627 |
| 1 | 33 (72.2%) | 100 (82.4%) |  |
| Disease stage |  |  |  |
| IIIB/IIIC | 5 (11.1%) | 23 (16.2%) | 0.423 |
| IV | 40 (88.9%) | 107 (83.8%) |  |
| Brain metastases at enrollment^*^ |  |  |  |
| Yes | 1 (0.0%) | 4 (4.4%) | 0.824 |
| No | 44 (100%) | 126 (95.6%) |  |
| PD-L1 tumor proportion score | | |  |
| <1% | 10 (33.3%) | 31 (22.1%) | 0.825 |
| ≥1% | 35 (66.7%) | 99 (77.9%) |  |

ECOG, Eastern Cooperative Oncology Group performance status; PD-(L)1, programmed cell death (ligand) 1.

## Supplemental Table S3: Summary of patients with adequate tumor tissues for mIF test.

|  | **Total** |  |
| --- | --- | --- |
|  | **(n=144)** |  |
| Age |  |  |
| ≥65 years | 36 (25.0%) |  |
| <65 years | 108 (75.0%) |  |
| Sex |  |  |
| Female | 41 (28.5%) |  |
| Male | 103 (71.5%) |  |
| Smoking history |  |  |
| ≥400 cigarette-years | 96 (66.7%) |  |
| <400 cigarette-years or never | 48 (33.3%) |  |
| ECOG performance status | | |
| 0 | 39 (27.1%) |  |
| 1 | 105 (72.9%) |  |
| Disease stage |  |  |
| IIIB/IIIC | 26 (18.1%) |  |
| IV | 118 (81.9%) |  |
| Brain metastases at enrollment^*^ |  |  |
| Yes | 3 (2.1%) |  |
| No | 141 (97.9%) |  |
| PD-L1 tumor proportion score | | |
| <1% | 37 (25.7%) |  |
| ≥1% | 107 (74.3%) |  |

ECOG, Eastern Cooperative Oncology Group performance status; PD-(L)1, programmed cell death (ligand) 1.

## Supplemental Table S4: Original data of patients with adequate tumor tissues for mIF test.

| **Group** | **Age** | **Sex** | **Smoking history** | **ECOG PS** | **PD-L1 TPS** | **stage** | **RECIST best response** | CD4(Cell Density) | CD4(%) | PDL1(Cell Density) | PDL1(%) | CD68(Cell Density) | CD68(%) | FOXP3(Cell Density) | FOXP3(%) | CD8(Cell Density) | CD8(%) |
| --- | --- | --- | --- | --- | --- | --- | --- | --- | --- | --- | --- | --- | --- | --- | --- | --- | --- |
| PD-1 + chemo | 49 | Male | Never | 0 | 50% | III | PR | 167.3 | 1.9 | 8926.3 | 99.3 | 1902.1 | 21.2 | 33.1 | 0.4 | 2247.6 | 25.0 |
| Chemo | 60 | Male | Never | 0 | 55% | III | PR | 891.7 | 8.3 | 7269.2 | 67.8 | 166.6 | 1.6 | 14.0 | 0.1 | 86.8 | 0.8 |
| Chemo | 61 | Male | Never | 1 | 0% | III | SD | 208.9 | 2.6 | 3486.7 | 42.6 | 0.0 | 0.0 | 48.2 | 0.6 | 289.2 | 3.5 |
| PD-1 + chemo | 49 | Female | Never | 1 | 5% | III | PR | 57.5 | 0.8 | 1065.5 | 15.5 | 175.2 | 2.5 | 60.3 | 0.9 | 11.0 | 0.2 |
| PD-1 + chemo | 57 | Female | Never | 0 | 40% | III | PR | 748.8 | 10.4 | 7151.2 | 99.0 | 46.9 | 0.6 | 5.9 | 0.1 | 101.7 | 1.4 |
| Chemo | 50 | Female | Never | 1 | 35% | III | SD | 1816.2 | 36.2 | 2352.5 | 46.8 | 600.0 | 11.9 | 221.0 | 4.4 | 2300.4 | 45.8 |
| Chemo | 68 | Female | Never | 1 | 20% | III | PR | 85.2 | 3.0 | 877.5 | 31.0 | 233.3 | 8.2 | 3.7 | 0.1 | 25.9 | 0.9 |
| PD-1 + chemo | 54 | Male | Ever/current | 1 | 3% | III | PR | 216.0 | 2.7 | 4948.1 | 61.0 | 1029.6 | 12.7 | 93.0 | 1.1 | 2699.3 | 33.3 |
| PD-1 + chemo | 54 | Male | Ever/current | 0 | 70% | III | NE | 123.7 | 4.1 | 2496.5 | 83.0 | 0.0 | 0.0 | 0.0 | 0.0 | 38.6 | 1.3 |
| PD-1 + chemo | 57 | Male | Ever/current | 0 | 0% | III | PR | 856.8 | 8.5 | 3668.2 | 36.4 | 3813.1 | 37.8 | 493.5 | 4.9 | 1085.4 | 10.8 |
| PD-1 + chemo | 57 | Male | Ever/current | 0 | 1% | III | PR | 148.1 | 3.4 | 541.8 | 12.6 | 132.5 | 3.1 | 64.3 | 1.5 | 37.0 | 0.9 |
| PD-1 + chemo | 57 | Male | Ever/current | 1 | 1% | III | NE | 823.0 | 13.9 | 743.1 | 12.6 | 240.6 | 4.1 | 184.1 | 3.1 | 110.6 | 1.9 |
| PD-1 + chemo | 57 | Male | Ever/current | 1 | 15% | III | PR | 118.2 | 1.8 | 5090.1 | 75.6 | 336.0 | 5.0 | 28.7 | 0.4 | 665.2 | 9.9 |
| PD-1 + chemo | 58 | Male | Ever/current | 0 | 1% | III | SD | 0.0 | 0.0 | 279.3 | 3.5 | 27.9 | 0.3 | 0.0 | 0.0 | 0.0 | 0.0 |
| PD-1 + chemo | 63 | Male | Ever/current | 1 | 2% | III | PR | 377.0 | 6.9 | 906.6 | 16.5 | 320.9 | 5.9 | 1.4 | 0.0 | 231.0 | 4.2 |
| PD-1 + chemo | 63 | Male | Ever/current | 0 | 10% | III | PR | 145.4 | 3.0 | 2112.2 | 43.9 | 163.5 | 3.4 | 4.5 | 0.1 | 122.6 | 2.5 |
| PD-1 + chemo | 65 | Male | Ever/current | 1 | 0% | III | PR | 43.4 | 0.8 | 143.6 | 2.6 | 79.3 | 1.4 | 1.7 | 0.0 | 66.8 | 1.2 |
| PD-1 + chemo | 65 | Male | Ever/current | 1 | 3% | III | PR | 144.9 | 3.8 | 1284.6 | 34.0 | 99.8 | 2.6 | 215.7 | 5.7 | 154.5 | 4.1 |
| Chemo | 52 | Male | Ever/current | 0 | 0% | III | SD | 1871.2 | 39.6 | 99.9 | 2.1 | 68.1 | 1.4 | 149.9 | 3.2 | 208.9 | 4.4 |
| Chemo | 56 | Male | Ever/current | 1 | 2% | III | PR | 2.3 | 0.1 | 823.1 | 18.9 | 664.7 | 15.3 | 0.0 | 0.0 | 371.1 | 8.5 |
| Chemo | 57 | Male | Ever/current | 1 | 0% | III | SD | 53.1 | 1.0 | 12.5 | 0.2 | 36.2 | 0.6 | 5.9 | 0.1 | 90.0 | 1.6 |
| Chemo | 58 | Male | Ever/current | 1 | 80% | III | PR | 17.9 | 0.4 | 3084.5 | 74.1 | 204.4 | 4.9 | 558.2 | 13.4 | 596.4 | 14.3 |
| Chemo | 60 | Male | Ever/current | 1 | 75% | III | PR | 707.8 | 15.7 | 1824.9 | 40.6 | 326.5 | 7.3 | 0.8 | 0.0 | 10.1 | 0.2 |
| Chemo | 63 | Male | Ever/current | 1 | 5% | III | PR | 169.3 | 4.2 | 251.4 | 6.2 | 259.0 | 6.4 | 26.2 | 0.7 | 173.5 | 4.3 |
| PD-1 + chemo | 62 | Female | Ever/current | 1 | 10% | III | PR | 623.2 | 7.7 | 102.2 | 1.3 | 437.8 | 5.4 | 221.6 | 2.7 | 366.2 | 4.5 |
| PD-1 + chemo | 46 | Male | Never | 1 | 1% | IV | SD | 42.3 | 0.6 | 1090.1 | 15.8 | 9.1 | 0.1 | 3.0 | 0.0 | 0.0 | 0.0 |
| Chemo | 43 | Male | Never | 1 | 1% | IV | PR | 9.8 | 0.2 | 312.2 | 4.8 | 35.0 | 0.5 | 0.0 | 0.0 | 8.9 | 0.1 |
| Chemo | 43 | Male | Never | 1 | 45% | IV | SD | 117.5 | 3.2 | 3245.9 | 87.2 | 74.2 | 2.0 | 5.3 | 0.1 | 3.1 | 0.1 |
| Chemo | 62 | Male | Never | 1 | 1% | IV | PR | 966.5 | 16.1 | 3038.7 | 50.5 | 427.3 | 7.1 | 4.3 | 0.1 | 1.3 | 0.0 |
| Chemo | 65 | Male | Never | 0 | 0% | IV | SD | 177.9 | 2.1 | 2082.5 | 24.5 | 514.8 | 6.0 | 0.0 | 0.0 | 210.0 | 2.5 |
| Chemo | 65 | Male | Never | 1 | 10% | IV | PD | 95.8 | 2.3 | 1571.0 | 38.5 | 218.2 | 5.3 | 6.7 | 0.2 | 20.0 | 0.5 |
| Chemo | 69 | Male | Never | 1 | 0% | IV | UN | 494.0 | 7.2 | 0.0 | 0.0 | 978.0 | 14.3 | 5.0 | 0.1 | 17.5 | 0.3 |
| PD-1 + chemo | 32 | Female | Never | 0 | 55% | IV | PR | 134.2 | 1.7 | 7282.3 | 89.8 | 140.8 | 1.7 | 113.0 | 1.4 | 48.5 | 0.6 |
| PD-1 + chemo | 40 | Female | Never | 0 | 80% | IV | PR | 474.5 | 8.3 | 3578.7 | 62.3 | 501.5 | 8.7 | 100.3 | 1.7 | 47.0 | 0.8 |
| PD-1 + chemo | 47 | Female | Never | 1 | 25% | IV | PR | 306.3 | 3.5 | 2410.5 | 27.5 | 839.1 | 9.6 | 94.2 | 1.1 | 65.2 | 0.7 |
| PD-1 + chemo | 48 | Female | Never | 1 | 3% | IV | PR | 318.9 | 6.1 | 2398.1 | 46.1 | 61.3 | 1.2 | 12.3 | 0.2 | 2.0 | 0.0 |
| PD-1 + chemo | 49 | Female | Never | 0 | 25% | IV | PR | 220.8 | 3.2 | 2999.0 | 43.7 | 262.3 | 3.8 | 10.5 | 0.2 | 126.6 | 1.8 |
| PD-1 + chemo | 52 | Female | Never | 1 | 5% | IV | PR | 1207.8 | 24.0 | 1045.0 | 20.8 | 350.4 | 7.0 | 41.9 | 0.8 | 199.9 | 4.0 |
| PD-1 + chemo | 53 | Female | Never | 1 | 2% | IV | PR | 1721.1 | 30.5 | 669.1 | 11.9 | 312.6 | 5.5 | 25.1 | 0.4 | 145.8 | 2.6 |
| PD-1 + chemo | 54 | Female | Never | 0 | 5% | IV | SD | 1991.0 | 24.0 | 402.4 | 4.9 | 356.5 | 4.3 | 22.9 | 0.3 | 33.4 | 0.4 |
| PD-1 + chemo | 54 | Female | Never | 1 | 80% | IV | PR | 202.9 | 4.2 | 4463.8 | 92.8 | 58.0 | 1.2 | 0.0 | 0.0 | 144.9 | 3.0 |
| PD-1 + chemo | 54 | Female | Never | 0 | NA | IV | PR | 271.4 | 5.7 | 12.9 | 0.3 | 0.0 | 0.0 | 6.5 | 0.1 | 34.5 | 0.7 |
| PD-1 + chemo | 57 | Female | Never | 1 | 20% | IV | PR | 420.4 | 9.3 | 4024.2 | 88.5 | 280.3 | 6.2 | 30.0 | 0.7 | 70.1 | 1.5 |
| PD-1 + chemo | 59 | Female | Never | 1 | 0% | IV | SD | 60.1 | 0.9 | 90.5 | 1.4 | 60.9 | 0.9 | 21.4 | 0.3 | 63.5 | 1.0 |
| PD-1 + chemo | 59 | Female | Never | 1 | 1% | IV | PR | 934.4 | 14.1 | 109.0 | 1.6 | 88.0 | 1.3 | 13.8 | 0.2 | 61.4 | 0.9 |
| PD-1 + chemo | 62 | Female | Never | 1 | 1% | IV | PR | 4.3 | 0.1 | 990.9 | 16.8 | 354.6 | 6.0 | 33.9 | 0.6 | 135.6 | 2.3 |
| PD-1 + chemo | 62 | Female | Never | 1 | 15% | IV | PR | 3638.4 | 69.4 | 1422.5 | 27.1 | 465.1 | 8.9 | 41.0 | 0.8 | 1758.6 | 33.5 |
| PD-1 + chemo | 63 | Female | Never | 1 | 35% | IV | PR | 405.9 | 5.1 | 2839.4 | 35.9 | 42.0 | 0.5 | 25.6 | 0.3 | 2.1 | 0.0 |
| PD-1 + chemo | 64 | Female | Never | 1 | 0% | IV | PR | 5.2 | 0.1 | 5.2 | 0.1 | 222.5 | 3.4 | 0.0 | 0.0 | 113.8 | 1.7 |
| PD-1 + chemo | 68 | Female | Never | 1 | 0% | IV | PR | 3.9 | 0.1 | 78.2 | 1.2 | 44.3 | 0.7 | 447.6 | 7.0 | 16.3 | 0.3 |
| PD-1 + chemo | 69 | Female | Never | 0 | 2% | IV | SD | 1515.5 | 14.4 | 1240.1 | 11.8 | 460.3 | 4.4 | 367.3 | 3.5 | 1095.6 | 10.4 |
| Chemo | 50 | Female | Never | 1 | 3% | IV | SD | 111.3 | 1.6 | 3879.3 | 56.1 | 267.8 | 3.9 | 16.8 | 0.2 | 251.5 | 3.6 |
| Chemo | 51 | Female | Never | 0 | 30% | IV | SD | 1736.4 | 16.8 | 5514.0 | 53.2 | 1048.7 | 10.1 | 35.2 | 0.3 | 9.5 | 0.1 |
| Chemo | 53 | Female | Never | 1 | 0% | IV | SD | 1670.2 | 32.2 | 732.7 | 14.1 | 572.0 | 11.0 | 21.2 | 0.4 | 39.1 | 0.8 |
| Chemo | 55 | Female | Never | 1 | 1% | IV | NE | 378.0 | 7.0 | 2075.5 | 38.2 | 94.2 | 1.7 | 7.7 | 0.1 | 72.1 | 1.3 |
| Chemo | 58 | Female | Never | 1 | 20% | IV | PR | 1437.0 | 38.8 | 3420.7 | 92.4 | 343.6 | 9.3 | 62.5 | 1.7 | 1030.9 | 27.8 |
| Chemo | 59 | Female | Never | 0 | 0% | IV | PR | 104.0 | 1.3 | 2918.2 | 37.3 | 624.9 | 8.0 | 123.4 | 1.6 | 583.0 | 7.5 |
| Chemo | 60 | Female | Never | 1 | 0% | IV | SD | 1051.8 | 10.7 | 546.3 | 5.6 | 70.0 | 0.7 | 218.2 | 2.2 | 98.5 | 1.0 |
| Chemo | 61 | Female | Never | 1 | 1% | IV | UN | 784.6 | 9.4 | 735.4 | 8.8 | 42.3 | 0.5 | 0.5 | 0.0 | 19.0 | 0.2 |
| Chemo | 62 | Female | Never | 1 | 0% | IV | SD | 232.0 | 3.7 | 711.6 | 11.3 | 108.2 | 1.7 | 1.7 | 0.0 | 0.0 | 0.0 |
| Chemo | 64 | Female | Never | 1 | 0% | IV | SD | 80.9 | 1.1 | 242.8 | 3.3 | 23.1 | 0.3 | 34.7 | 0.5 | 138.7 | 1.9 |
| Chemo | 65 | Female | Never | 1 | 0% | IV | PR | 160.1 | 1.7 | 396.9 | 4.2 | 32.2 | 0.3 | 389.1 | 4.1 | 287.9 | 3.0 |
| Chemo | 66 | Female | Never | 0 | NA | IV | SD | 430.3 | 5.3 | 1158.9 | 14.3 | 405.3 | 5.0 | 525.4 | 6.5 | 72.5 | 0.9 |
| Chemo | 67 | Female | Never | 0 | 2% | IV | PR | 627.1 | 9.7 | 2171.1 | 33.6 | 14.5 | 0.2 | 155.9 | 2.4 | 83.4 | 1.3 |
| Chemo | 67 | Female | Never | 1 | 10% | IV | PR | 1522.8 | 14.7 | 8737.7 | 84.1 | 1837.1 | 17.7 | 3212.2 | 30.9 | 0.0 | 0.0 |
| Chemo | 68 | Female | Never | 0 | 3% | IV | SD | 1058.0 | 14.5 | 149.5 | 2.1 | 3.9 | 0.1 | 5.2 | 0.1 | 37.7 | 0.5 |
| PD-1 + chemo | 38 | Male | Ever/current | 0 | 0% | IV | PR | 1343.0 | 16.0 | 468.3 | 5.6 | 1727.0 | 20.6 | 192.9 | 2.3 | 279.1 | 3.3 |
| PD-1 + chemo | 48 | Male | Ever/current | 1 | 0% | IV | PD | 145.7 | 2.5 | 623.4 | 10.7 | 340.9 | 5.8 | 639.0 | 10.9 | 69.5 | 1.2 |
| PD-1 + chemo | 50 | Male | Ever/current | 1 | 35% | IV | PR | 184.4 | 3.8 | 2670.5 | 55.3 | 400.8 | 8.3 | 14.0 | 0.3 | 1.4 | 0.0 |
| PD-1 + chemo | 52 | Male | Ever/current | 1 | 0% | IV | PD | 0.0 | 0.0 | 1766.3 | 30.6 | 185.9 | 3.2 | 10.9 | 0.2 | 0.0 | 0.0 |
| PD-1 + chemo | 53 | Male | Ever/current | 1 | 0% | IV | SD | 27.2 | 0.4 | 19.4 | 0.3 | 40.7 | 0.5 | 40.7 | 0.5 | 48.5 | 0.6 |
| PD-1 + chemo | 54 | Male | Ever/current | 0 | 0% | IV | PR | 84.7 | 1.0 | 1332.4 | 15.2 | 233.4 | 2.7 | 237.1 | 2.7 | 2343.0 | 26.7 |
| PD-1 + chemo | 54 | Male | Ever/current | 0 | 65% | IV | PR | 36.2 | 0.6 | 2569.1 | 42.6 | 15.7 | 0.3 | 53.4 | 0.9 | 12.4 | 0.2 |
| PD-1 + chemo | 55 | Male | Ever/current | 1 | 2% | IV | SD | 1146.6 | 17.0 | 664.4 | 9.9 | 476.9 | 7.1 | 69.7 | 1.0 | 584.0 | 8.7 |
| PD-1 + chemo | 55 | Male | Ever/current | 1 | 75% | IV | PR | 139.5 | 2.0 | 3392.6 | 49.8 | 57.0 | 0.8 | 15.9 | 0.2 | 11.1 | 0.2 |
| PD-1 + chemo | 56 | Male | Ever/current | 1 | 3% | IV | PR | 1056.9 | 12.9 | 2026.3 | 24.7 | 629.0 | 7.7 | 477.7 | 5.8 | 507.6 | 6.2 |
| PD-1 + chemo | 57 | Male | Ever/current | 1 | 25% | IV | SD | 133.9 | 2.1 | 1244.4 | 19.9 | 912.4 | 14.6 | 274.0 | 4.4 | 2067.7 | 33.0 |
| PD-1 + chemo | 58 | Male | Ever/current | 1 | 0% | IV | SD | 288.8 | 5.5 | 495.1 | 9.4 | 123.8 | 2.4 | 0.0 | 0.0 | 0.0 | 0.0 |
| PD-1 + chemo | 58 | Male | Ever/current | 0 | 2% | IV | PR | 1055.3 | 15.1 | 2291.7 | 32.8 | 453.6 | 6.5 | 260.7 | 3.7 | 347.1 | 5.0 |
| PD-1 + chemo | 58 | Male | Ever/current | 0 | 3% | IV | PR | 45.7 | 0.6 | 3.3 | 0.0 | 0.0 | 0.0 | 13.1 | 0.2 | 29.4 | 0.4 |
| PD-1 + chemo | 59 | Male | Ever/current | 1 | 0% | IV | PR | 62.7 | 1.3 | 4138.8 | 84.2 | 1120.4 | 22.8 | 4.2 | 0.1 | 175.6 | 3.6 |
| PD-1 + chemo | 59 | Male | Ever/current | 0 | 3% | IV | PR | 388.6 | 6.1 | 347.4 | 5.4 | 334.8 | 5.2 | 16.1 | 0.3 | 34.3 | 0.5 |
| PD-1 + chemo | 59 | Male | Ever/current | 1 | 10% | IV | NE | 31.5 | 0.4 | 3909.8 | 55.0 | 102.3 | 1.4 | 15.7 | 0.2 | 94.4 | 1.3 |
| PD-1 + chemo | 60 | Male | Ever/current | 0 | 0% | IV | SD | 81.3 | 0.9 | 116.7 | 1.3 | 43.5 | 0.5 | 12.9 | 0.1 | 11.1 | 0.1 |
| PD-1 + chemo | 60 | Male | Ever/current | 1 | 0% | IV | SD | 65.2 | 1.1 | 58.4 | 1.0 | 67.4 | 1.1 | 29.4 | 0.5 | 166.0 | 2.7 |
| PD-1 + chemo | 60 | Male | Ever/current | 1 | 0% | IV | PD | 179.5 | 3.0 | 10.3 | 0.2 | 150.1 | 2.5 | 72.1 | 1.2 | 0.0 | 0.0 |
| PD-1 + chemo | 60 | Male | Ever/current | 1 | 5% | IV | NE | 0.0 | 0.0 | 2808.9 | 40.1 | 28.1 | 0.4 | 0.0 | 0.0 | 0.0 | 0.0 |
| PD-1 + chemo | 61 | Male | Ever/current | 1 | 1% | IV | SD | 1434.4 | 15.5 | 1598.6 | 17.3 | 701.5 | 7.6 | 46.8 | 0.5 | 686.1 | 7.4 |
| PD-1 + chemo | 61 | Male | Ever/current | 1 | 1% | IV | UN | 14.1 | 0.2 | 0.0 | 0.0 | 425.9 | 6.7 | 2.8 | 0.0 | 0.0 | 0.0 |
| PD-1 + chemo | 61 | Male | Ever/current | 1 | 1% | IV | SD | 8.2 | 0.2 | 53.5 | 1.3 | 12.4 | 0.3 | 20.6 | 0.5 | 45.3 | 1.1 |
| PD-1 + chemo | 61 | Male | Ever/current | 1 | 2% | IV | SD | 4.3 | 0.1 | 482.9 | 8.6 | 150.0 | 2.7 | 22.0 | 0.4 | 37.2 | 0.7 |
| PD-1 + chemo | 61 | Male | Ever/current | 0 | 55% | IV | PD | 664.8 | 10.9 | 4533.0 | 74.1 | 439.6 | 7.2 | 3.3 | 0.1 | 12.4 | 0.2 |
| PD-1 + chemo | 62 | Male | Ever/current | 1 | 2% | IV | SD | 5.9 | 0.1 | 92.1 | 2.2 | 20.8 | 0.5 | 1.5 | 0.0 | 0.0 | 0.0 |
| PD-1 + chemo | 62 | Male | Ever/current | 1 | 35% | IV | SD | 163.0 | 1.8 | 4829.5 | 53.1 | 18.1 | 0.2 | 6.0 | 0.1 | 39.2 | 0.4 |
| PD-1 + chemo | 63 | Male | Ever/current | 1 | 75% | IV | PR | 59.7 | 1.0 | 4912.9 | 82.5 | 294.3 | 4.9 | 0.0 | 0.0 | 721.1 | 12.1 |
| PD-1 + chemo | 64 | Male | Ever/current | 1 | 0% | IV | PD | 1450.1 | 15.3 | 321.1 | 3.4 | 394.9 | 4.2 | 104.0 | 1.1 | 1112.5 | 11.7 |
| PD-1 + chemo | 64 | Male | Ever/current | 1 | 2% | IV | PR | 124.2 | 1.8 | 295.4 | 4.4 | 153.2 | 2.3 | 23.5 | 0.3 | 197.4 | 2.9 |
| PD-1 + chemo | 64 | Male | Ever/current | 0 | 3% | IV | PR | 1217.2 | 21.6 | 1.0 | 0.0 | 776.1 | 13.8 | 121.4 | 2.2 | 129.1 | 2.3 |
| PD-1 + chemo | 64 | Male | Ever/current | 1 | 15% | IV | SD | 14.0 | 0.3 | 76.2 | 1.5 | 6.3 | 0.1 | 58.6 | 1.2 | 145.5 | 2.9 |
| PD-1 + chemo | 66 | Male | Ever/current | 0 | 5% | IV | SD | 4185.6 | 46.0 | 89.0 | 1.0 | 453.0 | 5.0 | 409.7 | 4.5 | 2880.3 | 31.6 |
| PD-1 + chemo | 66 | Male | Ever/current | 1 | 45% | IV | SD | 2274.3 | 34.7 | 3106.2 | 47.5 | 876.8 | 13.4 | 102.1 | 1.6 | 1430.1 | 21.8 |
| PD-1 + chemo | 67 | Male | Ever/current | 1 | 0% | IV | SD | 247.4 | 5.9 | 1048.4 | 25.2 | 243.3 | 5.8 | 4.1 | 0.1 | 38.5 | 0.9 |
| PD-1 + chemo | 67 | Male | Ever/current | 0 | 2% | IV | PR | 126.1 | 1.5 | 0.8 | 0.0 | 45.3 | 0.6 | 21.0 | 0.3 | 654.1 | 8.0 |
| PD-1 + chemo | 67 | Male | Ever/current | 1 | 10% | IV | SD | 0.0 | 0.0 | 180.9 | 40.0 | 0.0 | 0.0 | 0.0 | 0.0 | 180.9 | 40.0 |
| PD-1 + chemo | 69 | Male | Ever/current | 1 | 20% | IV | PR | 932.3 | 14.1 | 4721.9 | 71.2 | 1053.4 | 15.9 | 12.1 | 0.2 | 835.4 | 12.6 |
| PD-1 + chemo | 69 | Male | Ever/current | 1 | 25% | IV | PR | 120.6 | 1.5 | 2565.3 | 31.3 | 211.0 | 2.6 | 9.8 | 0.1 | 112.6 | 1.4 |
| PD-1 + chemo | 69 | Male | Ever/current | 1 | 65% | IV | PR | 0.0 | 0.0 | 7980.4 | 99.2 | 111.4 | 1.4 | 19.4 | 0.2 | 256.8 | 3.2 |
| Chemo | 31 | Male | Ever/current | 1 | 0% | IV | PR | 292.6 | 4.2 | 1048.2 | 15.2 | 292.6 | 4.2 | 1.5 | 0.0 | 281.0 | 4.1 |
| Chemo | 40 | Male | Ever/current | 0 | 1% | IV | SD | 301.6 | 4.9 | 554.9 | 8.9 | 159.3 | 2.6 | 8.9 | 0.1 | 29.5 | 0.5 |
| Chemo | 44 | Male | Ever/current | 1 | 40% | IV | PR | 105.7 | 2.8 | 1138.1 | 30.6 | 116.6 | 3.1 | 6.4 | 0.2 | 55.0 | 1.5 |
| Chemo | 47 | Male | Ever/current | 0 | 1% | IV | PR | 160.4 | 3.0 | 116.1 | 2.2 | 88.6 | 1.7 | 6.3 | 0.1 | 4.2 | 0.1 |
| Chemo | 47 | Male | Ever/current | 1 | 15% | IV | SD | 164.7 | 2.6 | 3519.5 | 55.9 | 180.2 | 2.9 | 101.3 | 1.6 | 70.9 | 1.1 |
| Chemo | 49 | Male | Ever/current | 1 | 50% | IV | PD | 367.6 | 5.7 | 4838.9 | 74.6 | 339.3 | 5.2 | 100.9 | 1.6 | 263.7 | 4.1 |
| Chemo | 49 | Male | Ever/current | 0 | NA | IV | SD | 674.9 | 13.8 | 1434.7 | 29.3 | 45.3 | 0.9 | 3.8 | 0.1 | 74.3 | 1.5 |
| Chemo | 53 | Male | Ever/current | 1 | 75% | IV | PR | 47.2 | 1.1 | 1197.5 | 28.8 | 217.7 | 5.2 | 44.4 | 1.1 | 70.1 | 1.7 |
| Chemo | 55 | Male | Ever/current | 1 | 0% | IV | SD | 14.7 | 0.5 | 942.6 | 32.7 | 0.0 | 0.0 | 14.7 | 0.5 | 0.0 | 0.0 |
| Chemo | 55 | Male | Ever/current | 1 | 50% | IV | PR | 53.2 | 0.7 | 3043.7 | 38.9 | 19.0 | 0.2 | 1.9 | 0.0 | 19.0 | 0.2 |
| Chemo | 55 | Male | Ever/current | 1 | NA | IV | SD | 956.3 | 16.0 | 1683.0 | 28.2 | 758.9 | 12.7 | 30.5 | 0.5 | 495.2 | 8.3 |
| Chemo | 57 | Male | Ever/current | 1 | 5% | IV | PD | 184.6 | 5.0 | 1212.5 | 33.1 | 544.6 | 14.9 | 39.0 | 1.1 | 123.3 | 3.4 |
| Chemo | 57 | Male | Ever/current | 1 | 20% | IV | PD | 1661.0 | 22.0 | 1744.0 | 23.1 | 476.7 | 6.3 | 206.8 | 2.7 | 1486.6 | 19.7 |
| Chemo | 61 | Male | Ever/current | 0 | 2% | IV | UN | 105.3 | 1.6 | 3308.9 | 50.7 | 52.7 | 0.8 | 193.1 | 3.0 | 359.9 | 5.5 |
| Chemo | 61 | Male | Ever/current | 1 | 3% | IV | PD | 125.7 | 1.5 | 512.6 | 6.1 | 260.4 | 3.1 | 68.8 | 0.8 | 3.0 | 0.0 |
| Chemo | 61 | Male | Ever/current | 0 | 20% | IV | PR | 603.8 | 8.8 | 4361.7 | 63.9 | 356.6 | 5.2 | 64.5 | 0.9 | 390.9 | 5.7 |
| Chemo | 61 | Male | Ever/current | 1 | 50% | IV | PD | 244.6 | 3.3 | 6066.7 | 81.9 | 630.3 | 8.5 | 118.2 | 1.6 | 392.3 | 5.3 |
| Chemo | 62 | Male | Ever/current | 1 | 0% | IV | SD | 479.1 | 8.1 | 3849.6 | 65.0 | 242.6 | 4.1 | 16.8 | 0.3 | 288.4 | 4.9 |
| Chemo | 62 | Male | Ever/current | 1 | 0% | IV | PR | 138.7 | 2.1 | 5541.3 | 82.0 | 773.3 | 11.4 | 226.5 | 3.4 | 652.9 | 9.7 |
| Chemo | 63 | Male | Ever/current | 1 | 1% | IV | SD | 875.7 | 13.2 | 5480.5 | 82.6 | 83.2 | 1.3 | 314.4 | 4.7 | 88.4 | 1.3 |
| Chemo | 65 | Male | Ever/current | 0 | 0% | IV | SD | 1081.7 | 19.8 | 66.3 | 1.2 | 126.1 | 2.3 | 19.2 | 0.4 | 209.5 | 3.8 |
| Chemo | 65 | Male | Ever/current | 1 | 10% | IV | PR | 542.6 | 8.1 | 2025.8 | 30.1 | 394.3 | 5.9 | 46.5 | 0.7 | 97.4 | 1.4 |
| Chemo | 65 | Male | Ever/current | 1 | 25% | IV | PD | 0.0 | 0.0 | 701.1 | 13.6 | 306.7 | 6.0 | 29.2 | 0.6 | 0.0 | 0.0 |
| Chemo | 66 | Male | Ever/current | 1 | 0% | IV | SD | 1074.4 | 13.4 | 805.8 | 10.0 | 817.2 | 10.2 | 295.1 | 3.7 | 87.0 | 1.1 |
| Chemo | 66 | Male | Ever/current | 1 | 0% | IV | PR | 711.8 | 12.3 | 2388.3 | 41.3 | 259.0 | 4.5 | 31.3 | 0.5 | 870.9 | 15.1 |
| Chemo | 66 | Male | Ever/current | 1 | 0% | IV | SD | 248.2 | 3.1 | 87.2 | 1.1 | 134.2 | 1.7 | 164.3 | 2.0 | 486.3 | 6.0 |
| Chemo | 66 | Male | Ever/current | 1 | 60% | IV | PR | 36.4 | 1.1 | 2216.5 | 65.5 | 21.1 | 0.6 | 3.8 | 0.1 | 28.8 | 0.9 |
| Chemo | 67 | Male | Ever/current | 1 | 1% | IV | PR | 190.5 | 3.0 | 502.9 | 7.9 | 100.2 | 1.6 | 9.0 | 0.1 | 30.7 | 0.5 |
| Chemo | 67 | Male | Ever/current | 0 | NA | IV | PR | 138.7 | 2.5 | 589.6 | 10.5 | 39.6 | 0.7 | 62.3 | 1.1 | 72.6 | 1.3 |
| Chemo | 68 | Male | Ever/current | 1 | 0% | IV | PD | 113.1 | 2.5 | 1957.1 | 43.8 | 139.2 | 3.1 | 26.1 | 0.6 | 0.0 | 0.0 |
| Chemo | 68 | Male | Ever/current | 1 | 1% | IV | SD | 13.9 | 0.4 | 2409.7 | 61.9 | 148.7 | 3.8 | 0.0 | 0.0 | 9.3 | 0.2 |
| Chemo | 68 | Male | Ever/current | 1 | 8% | IV | PD | 0.0 | 0.0 | 0.0 | 24.0 | 0.0 | 9.9 | 0.0 | 1.3 | 0.0 | 3.9 |
| Chemo | 69 | Male | Ever/current | 1 | 15% | IV | PD | 1325.0 | 22.5 | 456.6 | 7.8 | 23.5 | 0.4 | 60.2 | 1.0 | 1333.6 | 22.7 |
| Chemo | 69 | Male | Ever/current | 1 | 80% | IV | SD | 0.0 | 0.0 | 5905.6 | 96.1 | 216.6 | 3.5 | 0.0 | 0.0 | 36.1 | 0.6 |
| Chemo | 70 | Male | Ever/current | 1 | 1% | IV | SD | 21.4 | 0.4 | 213.9 | 3.7 | 604.3 | 10.4 | 0.0 | 0.0 | 74.9 | 1.3 |
| PD-1 + chemo | 35 | Female | Ever/current | 1 | 2% | IV | PR | 207.5 | 3.0 | 632.3 | 9.2 | 104.8 | 1.5 | 16.8 | 0.2 | 331.2 | 4.8 |
| Chemo | 58 | Female | Ever/current | 1 | NA | IV | PD | 187.4 | 2.7 | 3658.0 | 52.1 | 338.6 | 4.8 | 320.5 | 4.6 | 96.7 | 1.4 |
|  |  |  |  |  |  |  |  |  |  |  |  |  |  |  |  |  |  |

**References**

1. Chen S, Zhou Y, Chen Y and Gu J, fastp: an ultra-fast all-in-one FASTQ preprocessor. Bioinformatics (2018) 34: i884-i890. doi:10.1093/bioinformatics/bty560

2. Bolger AM, Lohse M and Usadel B, Trimmomatic: a flexible trimmer for Illumina sequence data. Bioinformatics (2014) 30: 2114-20. doi:10.1093/bioinformatics/btu170

3. Li H, Handsaker B, Wysoker A, Fennell T, Ruan J, Homer N, Marth G, Abecasis G, Durbin R and Genome Project Data Processing S, The Sequence Alignment/Map format and SAMtools. Bioinformatics (2009) 25: 2078-9. doi:10.1093/bioinformatics/btp352

4. McKenna A, Hanna M, Banks E, Sivachenko A, Cibulskis K, Kernytsky A, Garimella K, Altshuler D, Gabriel S, Daly M and DePristo MA, The Genome Analysis Toolkit: a MapReduce framework for analyzing next-generation DNA sequencing data. Genome Res (2010) 20: 1297-303. doi:10.1101/gr.107524.110

5. Cibulskis K, Lawrence MS, Carter SL, Sivachenko A, Jaffe D, Sougnez C, Gabriel S, Meyerson M, Lander ES and Getz G, Sensitive detection of somatic point mutations in impure and heterogeneous cancer samples. Nat Biotechnol (2013) 31: 213-9. doi:10.1038/nbt.2514

6. Wang K, Li M and Hakonarson H, ANNOVAR: functional annotation of genetic variants from high-throughput sequencing data. Nucleic Acids Res (2010) 38: e164. doi:10.1093/nar/gkq603

7. Wu F, Jiang T, Chen G, Huang Y, Zhou J, Lin L, Feng J, Wang Z, Shu Y, Shi J, Hu Y, Wang Q, Cheng Y, Chen J, Lin X, Wang Y, Huang J, Cui J, Cao L, Liu Y, Zhang Y, Pan Y, Zhao J, Wang L, Chang J, Chen Q, Ren X, Zhang W, Fan Y, He Z, Fang J, Gu K, Dong X, Zhang T, Shi W, Zou J, Bai X, Ren S, Zhou C and Came LSG, Multiplexed imaging of tumor immune microenvironmental markers in locally advanced or metastatic non-small-cell lung cancer characterizes the features of response to PD-1 blockade plus chemotherapy. Cancer Commun (Lond) (2022) 42: 1331-1346. doi:10.1002/cac2.12383

8. Chalmers ZR, Connelly CF, Fabrizio D, Gay L, Ali SM, Ennis R, Schrock A, Campbell B, Shlien A, Chmielecki J, Huang F, He Y, Sun J, Tabori U, Kennedy M, Lieber DS, Roels S, White J, Otto GA, Ross JS, Garraway L, Miller VA, Stephens PJ and Frampton GM, Analysis of 100,000 human cancer genomes reveals the landscape of tumor mutational burden. Genome Med (2017) 9: 34. doi:10.1186/s13073-017-0424-2

9. Kawaguchi S, Higasa K, Shimizu M, Yamada R and Matsuda F, HLA-HD: An accurate HLA typing algorithm for next-generation sequencing data. Hum Mutat (2017) 38: 788-797. doi:10.1002/humu.23230

10. Jurtz V, Paul S, Andreatta M, Marcatili P, Peters B and Nielsen M, NetMHCpan-4.0: Improved Peptide-MHC Class I Interaction Predictions Integrating Eluted Ligand and Peptide Binding Affinity Data. J Immunol (2017) 199: 3360-3368. doi:10.4049/jimmunol.1700893

11. Liu C, Yang X, Duffy B, Mohanakumar T, Mitra RD, Zody MC and Pfeifer JD, ATHLATES: accurate typing of human leukocyte antigen through exome sequencing. Nucleic Acids Res (2013) 41: e142. doi:10.1093/nar/gkt481
